# Reprogramming to restore youthful epigenetics of senescent nucleus pulposus cells for mitigating intervertebral disc degeneration and alleviating low back pain

**DOI:** 10.1038/s41413-025-00416-1

**Published:** 2025-03-12

**Authors:** Wenzheng Ma, Wantao Wang, Lei Zhao, Jinghao Fan, Lei Liu, Lin Huang, Baogan Peng, Jianru Wang, Baoshan Xu, Hongmei Liu, Decheng Wu, Zhaomin Zheng

**Affiliations:** 1 Department of Spine Surgery, The First Affiliated Hospital, Sun Yat-sen University, Guangzhou, 510080 China; 2 Pain Research Center, Sun Yat-sen University, Guangzhou, 510080 China; 3 Guangdong Provincial Key Laboratory of Advanced Biomaterials, Department of Biomedical Engineering, Southern University of Science and Technology, Shenzhen, 518055 China; 4 Department of Orthopedics, The Third Medical Centre of Chinese PLA General Hospital, Beijing, 100039 China; 5 Department of Spinal Surgery, Tianjin Hospital, Tianjin, 30021l China

**Keywords:** Diseases, Bone quality and biomechanics

## Abstract

Aging is a pivotal risk factor for intervertebral disc degeneration (IVDD) and chronic low back pain (LBP). The restoration of aging nucleus pulposus cells (NPCs) to a youthful epigenetic state is crucial for IVDD treatment, but remains a formidable challenge. Here, we proposed a strategy to partially reprogram and reinstate youthful epigenetics of senescent NPCs by delivering a plasmid carrier that expressed pluripotency-associated genes (*Oct4*, *Klf4* and *Sox2*) in Cavin2-modified exosomes (OKS@M-Exo) for treatment of IVDD and alleviating LBP. The functional OKS@M-Exo efficaciously alleviated senescence markers (*p16*^*INK4a*^, *p21*^*CIP1*^ and *p53*), reduced DNA damage and H4K20me3 expression, as well as restored proliferation ability and metabolic balance in senescent NPCs, as validated through in vitro experiments. In a rat model of IVDD, OKS@M-Exo maintained intervertebral disc height, nucleus pulposus hydration and tissue structure, effectively ameliorated IVDD via decreasing the senescence markers. Additionally, OKS@M-Exo reduced nociceptive behavior and downregulated nociception markers, indicating its efficiency in alleviating LBP. The transcriptome sequencing analysis also demonstrated that OKS@M-Exo could decrease the expression of age-related pathways and restore cell proliferation. Collectively, reprogramming by the OKS@M-Exo to restore youthful epigenetics of senescent NPCs may hold promise as a therapeutic platform to treat IVDD.

## Introduction

The nucleus pulposus cells (NPCs) play a pivotal role in maintaining health and function of intervertebral discs (IVD) in the spine.^1^ With aging or certain pathological conditions, NPCs can experience an increase in senescent cells population, which can result in various detrimental effects.^2,3^ One primary characteristic of senescence is a decline in NPCs’ self-renewal capacity.^4^ This diminished ability decreases cell proliferation and synthesis of new extracellular matrix (ECM) components, contributing to disc degeneration and loss of disc height. Aging NPCs often exhibit increased senescence which is a state of irreversible cell cycle arrest.^5^ Aging can also lead to changes in the phenotype of NPCs. Most cells undergoing senescence develop a senescence-associated secretory phenotype (SASP), contributing to local inflammation that impairs overall IVD health.^4–7^ Specifically, there may be a shift from a more matrix-producing phenotype towards a more catabolic phenotype with one typical characteristics of increased production of enzymes that degrade the ECM, such as matrix metalloproteinases (MMPs).^8^ Overall, accumulation of senescent NPCs contributes to the degenerative process of IVD, resulting in tissue dysfunction and potential spinal disorders. Therefore, effectively mitigating NPCs senescence within IVD may offer a promising approach for treating intervertebral disc degeneration (IVDD) and IVDD-associated low back pain (LBP).

Eepigenetic dysregulation is increasingly recognized as a fundamental hallmark of aging.^7,9^ During aging, epigenetics such as histone modification are dysregulated, while over expression of pluripotency-associated genes (*Oct4*, *Sox2*, *Klf4* and *c-Myc* [OSKM], also known as Yamanaka factors) can remodel these epigenetic markers.^10–13^ Multiple studies have shown that cell reprogramming through overexpression of OSKM can ameliorate senescent cell phenotypes.^12,14–16^ Yang et al. found that dysregulation of the epigenetics, cellular differentiation, and progression of the DNA methylation and senescence could be reversed by pluripotency-associated genes (*Oct4*, *Klf4* and *Sox2*, defined as OKS)-mediated rejuvenation.^17^ Ocampo et al. used the progeria mice crossed to mice carrying an OSKM polycystronic cassette (4 F) and a rtTA trans-activator and found that partial-reprogramming by short-term cyclic overexpression of OSKM ameliorated cellular and physiological hallmarks of aging and prolonged lifespan in the mouse model.^18^ Cheng et al. used the 4 F mice and found reprogramming through OSKM in vivo abrogates advancement of IVDD.^19^ Motivated by the above researches, reprogramming by overexpression of OKS to restore the youth epigenetics may hold a potential effect to retard senility of NPCs. Instead of using the transgenic animals^17–19^, adopting plasmids for topical application of gene therapy to reprogram epigenetics, may be also a viable treatment option due to its ease of handling and safety. However, improving the transfection efficiency of senescent NPCs is the key to gene therapy and still remains a challenge.

Currently, 37 gene therapy medicines that can treat many diseases in different fields, such as single-gene genetic diseases, cancer, and autoimmune diseases, have been approved for marketing worldwide.^20^ Plasmid DNA has many advantages of strong targeting, high controllability, few side effects, and therefore has attracted much attention in clinical practice.^20^ Some naked plasmid-based medicines are authorized to deal with limb ischemia or peripheral vascular disease.^21,22^ However, improving the transfection efficiency of plasmids to ensure sufficient gene expression remains a critical challenge in gene therapy applications. The safety profile and immune response associated with viral and nonviral vectors used for delivering therapeutic plasmid DNA continue to pose challenges in clinical settings.^23^ Therefore, an ideal vector for therapeutic plasmid DNA should efficiently deliver DNA to specific target cells without causing harm to the cells or host organism while avoiding triggering any immune responses. Exosomes are nano-sized vesicles secreted by cells containing various biomolecules such as proteins or nucleic acids.^24–28^ These exosomes possess unique properties including innate stability, low immunogenicity, and excellent tissue/cell penetration capacity which make them promising candidates as gene carriers.^29^ Considering these advantageous properties of exosomes, we propose utilizing exosomes as delivery vehicles for the OKS overexpression plasmid to potentially enhance transfection efficiency in senescent NPCs.

**Scheme 1:**
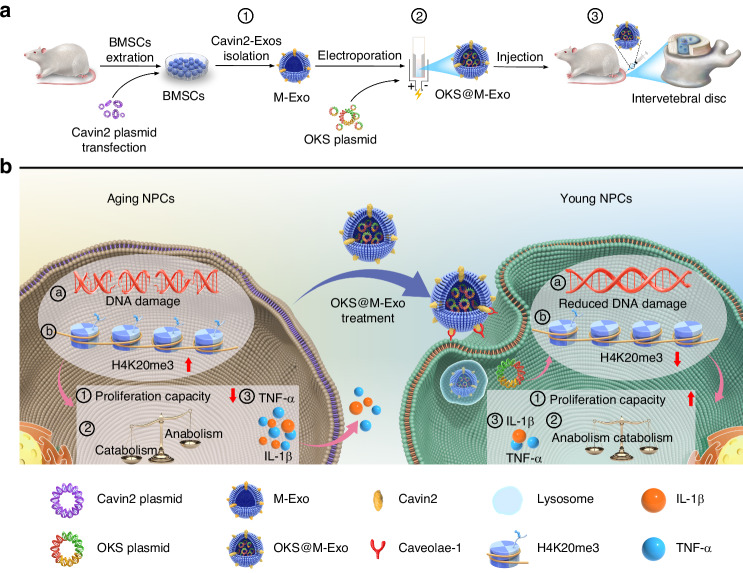
Schematic representations of OKS@M-Exo preparation and its application in IVDD. **a** Preparation and application of OKS@M-Exo. ① Cavin2 modified exosomes (M-Exo) were isolated from BMSCs transfected with the Cavin2 overexpression plasmid. ② The OKS plasmid was transfected into M-Exo using electroporation, resulting in OKS@M-Exo. ③ OKS@M-Exo was injected into the caudal IVD in rats. **b** OKS@M-Exo mitigated IVDD by restoring youthful epigenetic information in aging NPCs. OKS@M-Exo could reduced DNA damage ⓐ, and down-regulated the level of H4K20me3 ⓑ. OKS@M-Exo subsequently restored cell proliferation capacity ①, maintained the normal synthetic and catabolic metabolism balance of NPCs ②, and decreased the expression of inflammatory factors such as TNF-α and IL-1β ③

Here, we presented our findings on the surface modification of bone marrow mesenchymal stem cell (BMSCs)-derived exosomes with Cavin2 (M-Exo), which were used as carriers for delivering the OKS overexpression plasmid (OKS@M-Exo) to senescent NPCs, aiming to restore NPCs’ youthful epigenetic profile both in vitro and in vivo (Scheme 1). Initially, an OKS plasmid was successfully constructed and exhibited high expression level in senescent NPCs, leading to a reduced expression of age-related markers and enhancement of cell proliferation. The OKS plasmid restored the balance between synthetic and catabolic metabolism of NPCs. Subsequently, M-Exo were isolated from the Cavin2 modified BMSCs (M-BMSCs), serving as carriers for the OKS plasmid and facilitating enhanced uptake of senescent NPCs. Finally, in an extensive evaluation using a rat IVDD model, we observed that OKS@M-Exo effectively ameliorated senescence-related phenotypes, alleviated IVDD by maintaining disc height and NP tissue structure, and relieved LBP. Transcriptome sequencing analysis demonstrated that OKS@M-Exo reduced the expression of age-related pathways, restored cell proliferation and alleviated IVDD progression in rats. Our study may provide a novel target and tool for the treatment of IVDD or other aging-related disease.

## Results

### Aging-associated cellular phenotypes ameliorated by OKS in aging NPCs

**Fig. 1:**
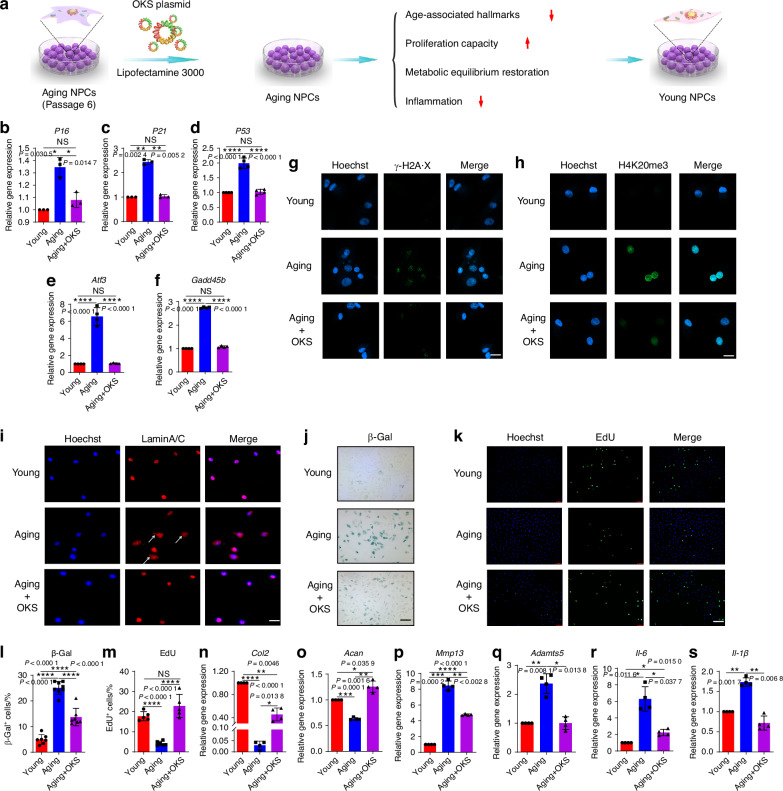
Partial expression of OKS ameliorated cellular phenotypes associated with aging and restored normal function of NPCs. **a** A schematic diagram illustrating the experimental design. The replicative senescence model of NPCs (passage 6, P6) was used to explore the physical function of OKS plasmids. RT-qPCR analysis of expression level of *P16* (**b**), *P21* (**c**), *P53* (**d**), *Atf3* (**e**) and *Gadd45b* (**f**) in Young group (passage 2 NPCs), Aging group (passage 6 NPCs) and Aging+OKS group (passage 6 NPCs treated with OKS plasmids). (*n* = 3/group). **g** Representative confocal immunofluorescence micrographs showing the expression of γ-H2A·X foci (green) in Young, Aging and Aging+OKS groups. Scale bar, 20 μm. **h** Representative confocal immunofluorescence micrographs showing the expression of H4K20me3 (green) in Young, Aging and Aging+OKS groups. Scale bar, 20 μm. **i** Representative immunofluorescence micrographs showing the expression Lamin A/C (red) of nuclear abnormality in Young, Aging and Aging+OKS groups. White arrows indicate blebbing in the nuclear envelope in cells. Scale bar, 50 μm. Representative images of β-Gal staining (**j**) and quantification (**l**) in Young, Aging and Aging+OKS groups. (*n* = 7-8/group). Scale bar, 100 μm. Representative immunofluorescence micrographs of EdU (**k**) and quantification (**m**) in Young, Aging and Aging+OKS groups. (*n* = 5/group). Scale bar, 100 μm. RT-qPCR analysis of senescence-associated anabolism factors *Col2* (**n**), *Acan* (**o**), and catabolism factors *Mmp13* (**p**), *Adamts5* (**q**) in Young, Aging and Aging+OKS groups. (*n* = 4/group). RT-qPCR analysis of senescence-associated inflammatory factors *Il-6* (**r**), *Il-1β* (**s**) in Young, Aging and Aging+OKS groups. (*n* = 3-4/group). *P < 0.05, **P < 0.01, ***P < 0.001, ****P < 0.000 1, NS means none sense, one-way ANOVA with Tukey’s multiple comparisons. Data are presented as the mean ± SD

To restore youthful epigenetics in senescent NPCs, we constructed a plasmid that expressed pluripotency-associated genes (OKS) to promote cellular reprogramming, activate pluripotency genes, and remodel the epigenome (Fig. 1a). First, we successfully constructed plasmid vectors that could simultaneously overexpress OKS, as verified by cutting the plasmid with a restriction endonuclease and analyzing the resulting fragments (Fig. S1a, b). The NPCs’ replicative senescence model (passage 6, P6) was used to investigate whether partial overexpression of OKS could promote regeneration of senescent NPCs. As shown in and Fig. S1c, d, the OKS were overexpressed in senescent NPCs after OKS plasmid transfection. The expression of *p16*^*INK4a*^ (*P16*), *p21*^*CIP1*^ (*P21*), *P53*, *Atf3* and *Gadd45b*, age-related stress response genes in the p53 tumor suppressor pathway, were downregulated in senescent NPCs after treatment of OKS plasmid (Fig. 1b–f). The number of foci for histone γ-H2A·X, a marker of nuclear DNA double-strand breaks associated with senescent^30^, was significantly reduced by partial expression of OKS compared with the aging NPCs (Fig. 1g). Increased histone H4K20 trimethylation (H4K20me3) and downregulated H3K9 trimethylation (H3K9me3) are two of the epigenetic marks associated with aging.^30–33^ As shown in Fig. 1h and Fig. S2, the expression of H4K20me3 was decreased and H3K9me3 was upregulated after OKS plasmid transfection compared with the aging NPCs. Lamins (mainly laminA/C) are components of the nuclear lamina, maintaining proper architecture of the nuclear envelope, and nuclear envelope abnormalities are associated with senescence.^7,34,35^ Importantly, over expression of OKS in senescent NPCs significantly decreased the blebbing in the nuclear envelope compared to aging cells, indicating OKS could retain nuclear envelope architecture (Fig. 1i). Furthermore, senescence associated β-galactosidase (SA-β-Gal) activity decreased in senescent NPCs after induction of OKS (Fig. 1j, l). Together, these results suggested that OKS plasmid ameliorated age-associated hallmarks in senescent NPCs.

A decline in self-renewal capacity is one of the main characteristics of NPCs senescence. By EdU assay, the percent of EdU^+^ cells in the Aging+OKS group significantly increased compared with the Aging group, indicating that overexpression of OKS could reverse the impaired proliferation capacity of aging NPCs (Fig. 1k, m). In addition, senescent NPCs may suffer from synthetic and catabolic disequilibrium. The partial overexpression of OKS in senescent NPCs promoted function recovery in senescent NPCs by boosting the expression of anabolism factor *Col2* and *Acan*, and downregulating the expression of catabolism factors *Mmp13* and *Adamts5* (Fig. 1n–q). The western blotting (WB) experiments further confirmed the downregulation of the expression of ADAMTs5 and MMP13 proteins after OKS treatment (Fig. S3a, b). By analyzing the immunofluorescence signals of ACAN and MMP13, we also observed an increase in ACAN expression and a decreased expression of MMP13 in the Aging+OKS group (Fig. S3c, d). These results further confirmed the ability of OKS to enhance function recovery in senescent NPCs. Senescent cells induce the formation of a complex, multicomponent SASP by secreting a range of cytokines and inflammatory factors. In the local microenvironment, the SASP alters the biological behavior of adjacent cells.^4,5,7^ Therefore, it is also necessary to find out whether OKS can reduce inflammation. As shown in Fig. 1r, s and Fig. S4a, b, partial expression of OKS could reduce the expression of IL-6, TNF-α and IL-1β compared with the Aging group. Collectively, these results showed that partial expression of OKS in senescent NPCs alleviated age-associated hallmarks, restored proliferation capacity and metabolic equilibrium, and decreased inflammation, indicating OKS could remodel the senescent process and promote a youthful state of NPCs.

### Cavin2-modified exosomes enhanced the uptake of OKS by aging NPCs

**Fig. 2:**
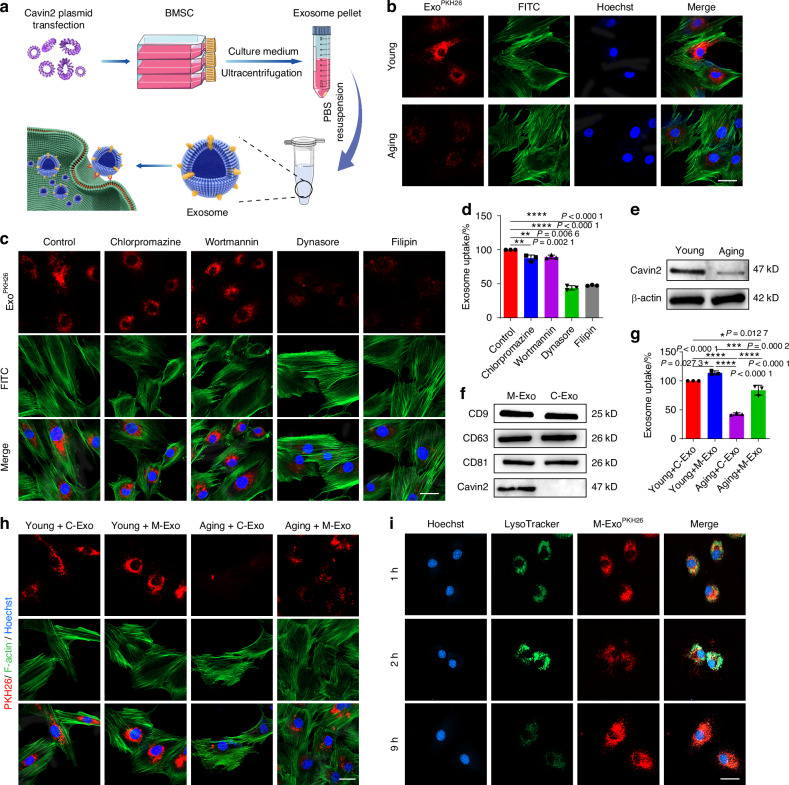
Cavin2-modified exosomes could be uptaken by aging NPCs via Caveolae-dependent endocytosis. **a** Schematic graph of the preparation for engineering exosomes. **b** Representative confocal fluorescence micrographs of Young and Aging NPCs, co-cultured with C-Exo. The exosomes were labeled with PKH26 (red) and the cytoskeleton was labeled with FITC (green). Scale bar, 20 μm. **c** NPCs were cultured under different inhibition conditions. Representative confocal fluorescence micrographs showed the internalization of PKH26-labeled (red) exosomes in these groups. Chlorpromazine: clathrin-mediated endocytosis inhibitor; Wortmannin: inhibitor of the phosphoinositide 3-kinase; Dynasore: dynamin inhibitor; Filipin: caveolae-dependent endocytosis inhibitor. Scale bar, 20 μm. **d** Fluorescence intensity of PKH26-labeled C-Exo taken up by NPCs under different inhibition conditions by flow cytometry (*n* = 3/group). **e** WB analysis of Cavin2 in Young and Aging NPCs. **f** WB analysis of CD9, CD63, CD81 and Cavin2 in C-Exo and M-Exo. **g** Fluorescence intensity of PKH26-labeled C-Exo or M-Exo taken up by Young or Aging NPCs by flow cytometry (*n* = 3/group). **h** Representative confocal fluorescence micrographs of PKH26-labeled C-Exo or M-Exo (red) taken up by Young or Aging NPCs. The cytoskeleton was labeled with FITC (green). Scale bar, 20 μm. **i** Representative confocal fluorescence micrographs of lysosomes (green) and exosomes (red) in NPCs co-cultured with exosomes for 1 h, 2 h and 9 h. Scale bar, 20 μm. *P < 0.05, **P < 0.01, ***P < 0.001, ****P < 0.000 1, one-way ANOVA with Tukey’s multiple comparisons. Data are presented as the mean ± SD

Low toxicity and high transfection efficiency are two of the most important factors for successful gene therapy.^23^ Exosomes represent a cutting-edge platform for targeted gene delivery, offering unique advantages such as stability, low immunogenicity, and efficient cell penetration.^29,36^ Therefore, we used BMSCs-derived exosomes as carriers to deliver OKS plasmid into senescent NPCs (Fig. 2a). BMSCs were obtained from the femur and tibial bone marrow of rats, and displayed spindle-like morphology (Fig. S5a). BMSCs are able to differentiate to osteocyte and adipocyte (Fig. S5b). The results of flow cytometry showed that BMSCs were positive for CD73, CD90 and CD105, and negative for HLA-DR, CD19 and CD11b (Fig. S5c). The above results suggested that BMSCs were successfully extracted. And then, the exosomes (C-Exo) were isolated from the BMSCs. Transmission electron microscopy (TEM) images showed that the majority of the particles exhibited a cup- or round-shaped morphology and the diameter of the exosomes was approximately 121 nm (Fig. S6a, b). As shown in Fig. 2b, we found that less exosomes taken up by the aging NPCs than the young ones. To guarantee that OKS can efficiently remodel the epigenetics of aging cells, it is crucial for enhancing the uptake efficiency of C-Exo in aging NPCs. The exosomes primarily gain access to NPCs through caveolae/lipid raft-dependent endocytosis.^37^ Our experimental results also demonstrated that C-Exo entered into NPCs through caveolae/lipid raft-dependent endocytosis (Fig. 2c, d and Fig. S7a). Cavin2 plays a crucial role in the formation of plasma membrane curvature and the endocytosis of extracellular cargoes by recruiting polymerase I and transcript release factor (PTRF) and binding to caveolin-1.^38^ The expression of Cavin2 decreased in aging NPCs (Fig. 2e), which may be the cause of the decline in phagocytic ability within the aging NPCs. So, to improve phagocytic ability, introduction of exogenous Cavin2 may provide a potential solution. Then, we produced exosomes with Cavin2 surface modification (M-Exo) through a three step strategy: (1) constructed Cavin2-Lamp2b overexpression plasmid (Fig. S5a, b), (2) transfected the plasmids into BMSCs, (3) isolated M-Exo from the culture medium of M-BMSCs. The morphology and size of the C-Exo and M-Exo were similar (Fig. S6). The results of WB analysis revealed robust expression of Cavin2 in M-Exo (Fig. S8c and Fig. 2f). To evaluate the uptake effects of M-Exo on aging NPCs, Paul Karl Horan 26 dye (PKH26) was used to label exosomes. Compared with the Aging + C-Exo group, the uptake of M-Exo increased in aging NPCs, suggesting that modified Cavin2 on exosomes enhanced the uptake of aging NPCs (Fig. 2h). For young NPCs, whether the exosomes were modified with Cavin2 or not, there was no difference in their ability to exosomes taken-up. The results of flow cytometry were consistence with the laser confocal detection (Fig. 2g and Fig. S7b). Lysosome escape is indeed crucial for nanoparticles to exert their cellular effects effectively. The fluorescence of PKH26 was completely overlapped with lysosomes after 1 h, partially out of alignment with lysosomal fluorescence after 2 h, and only a small portion of PKH26 fluorescence was overlapped with lysosomes after 9 h of incubation, indicating the exosomes effectively escaped from the lysosomes (Fig. 2i and Fig. S9). Taken together, these results indicated that the Cavin2 modified exosomes can be engulfed into senescent NPCs and escape from the lysosomes to function.

### OKS@M-Exo reduced the aging characteristics of senescent NPCs

**Fig. 3:**
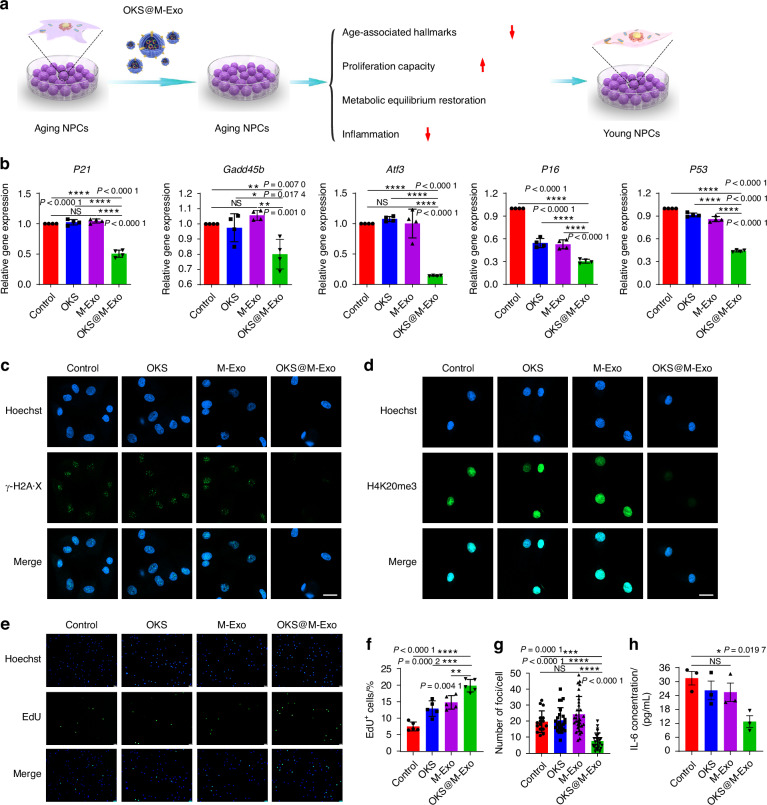
OKS@M-Exo mitigated the aging characteristics of aging NPCs. **a** A schematic diagram illustrating the experimental design. OKS plasmid transfected into the exosomes via electroporation. Different indicators, associated with aging and NPCs’ function, were detected. **b** RT-qPCR analysis of stress response genes in the P53 pathway including *P21*, *Gadd45b*, *Atf3*, *P16*, *P53* in aging NPCs treated with Control, OKS, M-Exo and OKS@M-Exo groups. (*n* = 4/group). **c, g** Representative confocal immunofluorescence micrographs showing the expression of γ-H2A·X foci (green) (**c**) and quantification (**g**) in aging NPCs treated with Control, OKS, M-Exo and OKS@M-Exo groups (*n* = 17–32/group). Scale bar, 20 μm. **d** Representative confocal immunofluorescence micrographs showing the expression of H4K20me3 (green) in aging NPCs treated with Control, OKS, M-Exo and OKS@M-Exo groups. Scale bar, 20 μm. Representative immunofluorescence micrographs of EdU (green) (**e**) and quantification (**f**) in aging NPCs treated with Control, OKS, M-Exo and OKS@M-Exo groups (*n* = 5/group). Scale bar, 100 μm. **h** The culture medium concentration of IL-6 in aging NPCs treated with Control, OKS, M-Exo and OKS@M-Exo groups were determined by ELISA assay (*n* = 3/group). *P < 0.05, **P < 0.01, ***P < 0.001, ****P < 0.000 1, NS means none sense, one-way ANOVA with Tukey’s multiple comparisons. Data are presented as the mean ± SD

We next assessed whether OKS@M-Exo treatment was able to restore youthful epigenetics of aging NPCs (Fig. 3a). First, we observed and analyzed the exosomes before and after electroporation through TEM and nanoparticle tracking analysis (NTA), and found that electroporation didn’t affect the morphology and size of the exosomes (Fig. S10a, b). To confirm OKS plasmids loaded into M-Exo via electroporation, we extracted OKS plasmids from OKS@M-Exo. Agarose Gel Electrophoresis was used to test the cut OKS plasmids by the restriction endonuclease. As shown in Fig. S10c, two fragments of OKS plasmid cut by restriction endonuclease were observed, indicating successful transfection of OKS plasmids into OKS@M-Exo. Moreover, the efficiency of loading OKS plasmids into exosomes by electroporation was approximately 35% (Fig. S10d).

The aging NPCs were used as Control group and treated with OKS plasmid (OKS group), M-Exo (M-Exo group) and OKS@M-Exo (OKS@M-Exo group) (Fig. 3). OKS@M-Exo has no effect on the proliferation of normal NPCs, suggesting that it’s a safe way of administration (Fig. S11a, b). As shown in and Fig. S12, the expressions of Oct4, Sox2, Klf4 proteins in aging NPCs increased after OKS@M-Exo treatment. Meanwhile, we performed a dual-luciferase reporter assay to evaluate the transcriptional activity of plasmids delivered into NPCs via exosomes. The results showed that both OKS plasmids (delivered with transfection reagent) and OKS@M-Exo treatment increased luciferase activity (Fig. S13). Compared with Control, OKS and M-Exo groups, the mRNA expression of *P21*, *Gadd45b*, *Atf3*, *P16* and *P53* was downregulated after OKS@M-Exo treatment (Fig. 3b). The number of foci for histone γ-H2A·X and the fluorescence intensity of H4K20me3 were significantly reduced by OKS@M-Exo treatment in aging NPCs compared with Control, OKS and M-Exo groups (Fig. 3c, d, g). And the expression of H3K9me3 was significantly increased in the OKS@M-Exo group compared with the other three groups (Fig. S14). These results suggested that OKS@M-Exo could ameliorate age-associated hallmarks. Meanwhile, OKS@M-Exo promoted the expression of ACAN and Col2, reduced the expression of MMP13 and ADAMTs5, and balanced anabolic and catabolic metabolism balance, thus restoring the normal structure of ECM (Figs. S15, S16). The proliferation of aging NPCs was rejuvenated after OKS@M-Exo treatment (Fig. 3e, f). Furthermore, the expression of inflammation-related factors, such as TNF-α, IL-1β and IL-6, were inhibited in aging NPCs after exposure to OKS@M-Exo (Fig. S17a, b and Fig. 3h). Altogether, these results showed that loading OKS into the exosomes could mitigate the aging characteristics of NPCs, and restore the metabolic balance and proliferative capacity of aging NPCs.

### OKS@M-Exo ameliorated IVDD and relieved LBP in vivo

**Fig. 4:**
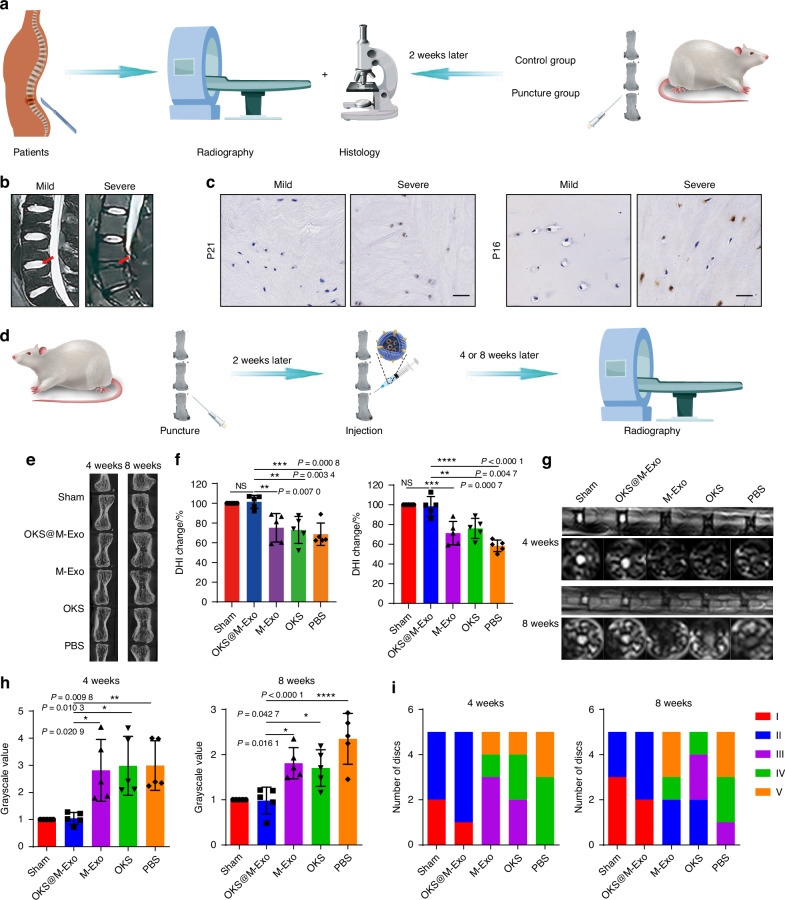
Radiological results of animal experiments indicated OKS@M-Exo could alleviate IVDD. **a** A schematic diagram illustrating the experimental design. Clinical patients conducted imaging examinations, and took the patients’ NP tissue specimens for histological testing during the operation. Imaging and histological tests were performed 2 weeks after the puncture of the rat tail spine. **b** Representative T2-weighted MRI scans of human lumbar spine (the red arrow indicated the IVD). **c** Representative immunohistochemical (IHC) analysis of human IVD sections to assess the expression of P16 and P21. Scale bar, 50 μm. **d** The schematic graph of IVDD modeling and the treatments. Different coccygeal IVD of each rat tail was divided into five groups for puncture and injection with various treatment groups. Radiological tests were performed at weeks 4 and 8 after treatment. Sham group: normal IVD (*n* = 5), PBS group: punctured IVD treated with PBS (*n* = 5), OKS group: punctured IVD treated with OKS plasmids (*n* = 5), M-Exo group: punctured IVD treated with M-Exo (*n* = 5), OKS@M-Exo group: punctured IVD treated with OKS@M-Exo (*n* = 5). **e** Representative Micro-CT images of coccygeal IVD in rats at weeks 4 and 8 after treatment. **f** The quantification of DHI changes in Sham, PBS, OKS, M-Exo and OKS@M-Exo groups at weeks 4 and 8 after treatment. (*n* = 5/group). **g** Representative T2-weighted MRI sagittal and cross-sectional images of coccygeal IVD in rats. **h** The quantification of gray values of coccygeal IVD in Sham, PBS, OKS, M-Exo, OKS@M-Exo groups at weeks 4 and 8 after treatment (*n* = 5/group). **i** The Pfirrmann classification of coccygeal IVD at weeks 4 and 8 after treatment to evaluate the degenerated level of IVD. (*n* = 5/group). *P < 0.05, **P < 0.01, ***P < 0.001, ****P < 0.000 1, NS means none sense, one-way ANOVA with Tukey’s multiple comparisons. Data are presented as the mean ± SD

We next assessed whether OKS@M-Exo treatment was able to restore youthful epigenetics of aging NPCs and mitigate IVDD. To investigate the therapeutic effects of OKS@M-Exo in vivo, a rat model of IVDD was created by inducing needle puncture in rat coccygeal discs. The needle puncture can simulate the pathological process of IVDD^39–41^, and also lead to signs of IVD aging.^42^ Imaging and histological tests were performed on clinical patients’ specimens and constructed rat IVDD models (Fig. 4a). The NP specimens were obtained from patients with mild and severe degeneration, demonstrated by magnetic resonance imaging (MRI) images (Fig. 4b), and detected the aging-related indicators including P21 and P16. As shown in Fig. 4c, the expression of P21 and P16 increased in severe degenerated IVD. The animal model was used to detect the degeneration and aging indicators of the rats’ caudal vertebra. After 2 weeks of puncture, the coccyx of the rats experienced IVDD, which verified by MRI, Micro-CT, hematoxylin-eosin (H&E) and Safranin O-Fast Green Staining (Safranin O) (Fig. S18a–c). Meanwhile, the expression of P21 increased, suggesting that the pathological process of IVDD could lead to senescence of NPCs in IVD (Fig. S18d). Due to IVDD and aging appeared 2 weeks after acupuncture of the coccygeal disc in rats, we performed drug administration 2 weeks after puncture to explore the therapeutic effect of OKS@M-Exo.

To verify that OKS@M-Exo can be taken up by NPCs, we locally injected either unlabeled or PKH26-labeled OKS@M-Exo (PKH26-Exo) into the IVD. As shown in Fig. S19, two days after the injection, OKS@M-Exo were mainly endocytosed by NPCs. Additionally, we tested the local expression levels of the three transcription factors. The results showed that the expression of Oct4, Klf4 and Sox2 in the OKS@M-Exo group was significantly higher than in the other four groups. (Fig. S20). Micro-CT and MRI were performed to measure the degree of IVDD at weeks 4 and 8 after injection of drugs (Fig. 4d). Disc height index (DHI), which refers to the variation of disc height, can reflect the degree of IVDD. A lower DHI indicates a greater degree of disc degeneration. Compared with PBS, OKS and M-Exo groups, the DHI in the OKS@M-Exo group was significantly improved and similar to that of the Sham group at weeks 4 and 8 after treatment (Fig. 4e, f). Subsequently, MRI scans were obtained to evaluate alterations in disc water content. This was analyzed using the grayscale values at the center of the discs, where higher grayscale values signify lower water content. There was a high T2-weighted signal intensity in sham group, and a low T2-weighted signal intensity in PBS group. As shown in Fig. 4g, the T2-weighted signal intensity of the OKS@M-Exo group was the highest among the PBS, OKS and M-Exo groups, similar to the Sham group. The OKS@M-Exo group had the lowest grayscale compared with PBS, OKS and M-Exo groups, suggesting that the water content of the OKS@M-Exo group was significantly restored (Fig. 4h). Pfirrmann grade, a semi-quantitative visual classification system, is currently recognized as a classification method for diagnosing the degree of IVDD.^43^ The Pfirrman MRI score of OKS@M-Exo group also remarkably decreased compared with PBS, OKS and M-Exo groups (Fig. 4i). These results suggested that OKS@M-Exo OKS@M-Exo had a better therapeutic effect on IVDD.

**Fig. 5:**
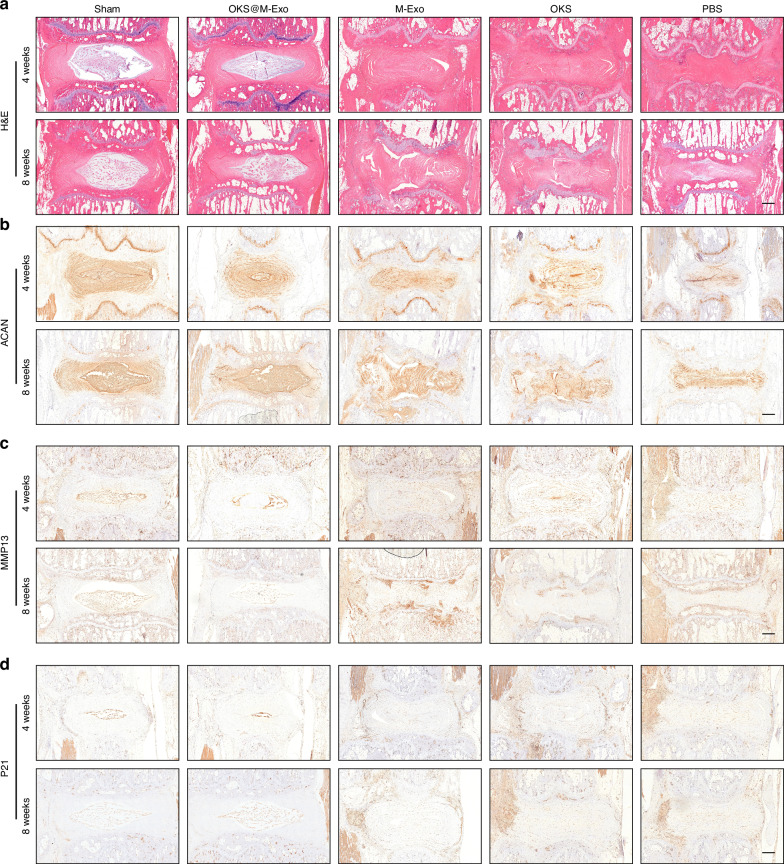
Histological results of animal experiments. **a** Representative H&E staining images of IVD in Sham, PBS, OKS, M-Exo and OKS@M-Exo groups at weeks 4 and 8 after treatment. Scale bar, 500 μm. Representative IHC analysis of rat IVD sections to assess ACAN (**b**), MMP13 (**c**), P21 (**d**) in Sham, PBS, OKS, M-Exo and OKS@M-Exo groups at weeks 4 and 8 after treatment. Scale bar, 500 μm

Histological analysis of the IVD in rats were conducted at weeks 4 and 8 after drug administration. The IVD in Sham and OKS@M-Exo groups showed a normal microstructure, in which the NP was intact and had a clear boundary with the AF. In PBS group, the NP was absent, and the IVD was disorganized. To the contrary, the IVD microstructure of OKS and M-Exo groups were not clear, but there was still some collagen in the disc tissue at weeks 4 and 8 after treatment (Fig. 5a and Fig. S21a). The histological score of OKS@M-Exo group was significantly lower than that of PBS, OKS, M-Exo groups, similar to that of Shame group at weeks 4 and 8 after treatment (Fig. S21b, c). The IHC results showed that Sham group and OKS@M-Exo groups had a stronger expression of ACAN and lower expression of MMP13, which were conducive to maintaining the normal structure and biomechanical properties of ECM. While the expression level of ACAN and MMP13 in OKS and M-Exo groups were not significantly different from those in PBS group (Fig. 5b, c). The results of Fig. 3 showed that OKS@M-Exo could ameliorate cellular phenotypes associated with aging. Therefore, we used IHC to detect the expression of aging-related indicators, and the results showed that OKS@M-Exo could downregulate the expression of P21 in the degenerative IVD tissue compared to PBS, OKS and M-Exo groups (Fig. 5d). Above all, these results suggested that OKS@M-Exo can maintain the normal structure and metabolic balance of IVD and reduce the expression of age-related factors, then play a role in alleviating IVDD.

**Fig. 6:**
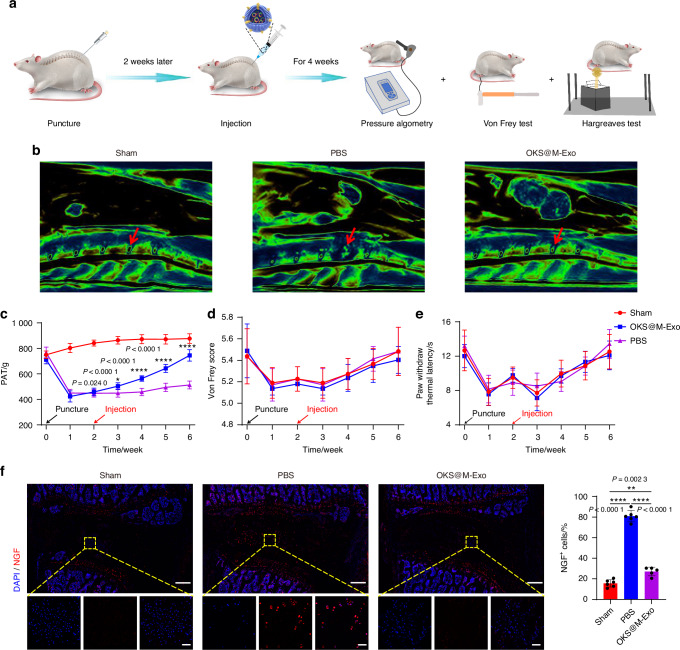
OKS@M-Exo effectively reduced the occurrence of LBP in the rat model of anterior lumbar puncture. **a** The schematic graph illustrated that OKS@M-Exo could alleviate LBP. The rats were divided into three groups, underwent anterior lumbar disc puncture (L4/5) and injection of different drugs to detect pain behavior. Sham group: normal IVD (*n* = 6), PBS group: punctured IVD treated with PBS (*n* = 6), OKS@M-Exo group: punctured IVD treated with OKS@M-Exo (*n* = 5). **b** Representative T2-weighted MRI sagittal images of lumbar IVD in rats (the red arrow indicated the IVD). **c** Temporal changes of PATs for the lumbar L4/5 regions in Sham, OKS@M-Exo and PBS groups. **d** Graph comparing the mechanical threshold (Von Frey test) in Sham, OKS@M-Exo and PBS groups. **e** Graph comparing the thermal withdrawal latency (Hargreaves test) in Sham, OKS@M-Exo and PBS groups. **f** Left: representative fluorescence images NGF (red) immunostaining at IVD. Yellow boxes indicate the area shown at ×30 magnification in images below. Right: quantification of NGF+ cells in Sham, OKS@M-Exo and PBS groups (*n* = 5 or 6/group). Scale bar, 500 μm (above), scale bar, 50 μm (below). *P < 0.05, **P < 0.01, ****P < 0.000 1, one-way ANOVA with Tukey’s multiple comparisons. Data are presented as the mean ± SD

We next examined the ability of OKS@M-Exo to relieve LBP in vivo. An animal model of anterior lumbar puncture in rat was constructed to assess the pain-associated behavioral changes (Fig. 6a). The rats were divided into three groups: Sham group, OKS@M-Exo group and PBS group. The results of MRI showed that OKS@M-Exo maintained the normal structure of IVD, indicating a better therapeutic effect on IVDD (Fig. 6b). Pressure algometry thresholds (PATs) was used to assess pain sensitivity and threshold levels in response to applied pressure (Fig. 6c). The baseline PATs of the Sham group (749.5 ± 27.6), OKS@M-Exo group (710.7 ± 29.1) and PBS group (767.4 ± 39.3) was similar at day 0 (without surgery). The PATs of the OKS@M-Exo group and the PBS group were significantly lower than that of the Sham group after weeks 1 and 2 post-surgery (without treatment), indicating successful induction of LBP. After the OKS@M-Exo treatment, we found that the PATs gradually increased and reached 744.8 ± 40.1 at weeks 6. Meanwhile, the PATs of the PBS treatment were tended to be stable with 513.2 ± 28.1 at weeks 6. The above results indicated that OKS@M-Exo could relieve back pain in rats (Fig. 6c). The increase of PATs in Sham group may be associated with the rise in animals’ body weight. The Von Frey and Hargreaves tests were conducted, which indicate radiation-induced leg pain. There was no significant difference between the Sham group, OKS@M-Exo group and PBS group (Fig. 6d, e). This lack of difference may be attributed to the fact that the anterior puncture did not rupture the posterior margin of the AF to form a disc herniation and did not stimulate the nerve roots to cause leg pain. During IVDD, nerve endings expand from the outer AF to the inner AF and NP regions of the disc, accompanied by increased levels of neurotrophic factors, such as nerve growth factor (NGF). These factors can lead to nerve sensitization.^6^ As shown in Fig. 6f, compared with the PBS group, the expression of NGF in IVD of OKS@M-Exo group decreased, indicating that nerve ingrowth reduced after OKS@M-Exo treatment and thereby alleviating LBP. Taken together, these results suggested that OKS@M-Exo can effectively mitigate IVDD and alleviate LBP.

### RNA-seq confirmed OKS@M-Exo alleviated the progression of IVDD

**Fig. 7:**
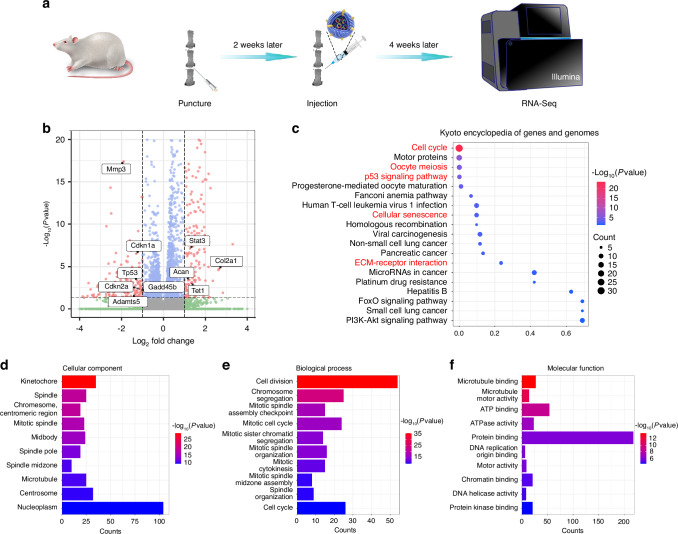
The RNA-seq analysis was performed between the OKS@M-Exo and PBS groups. **a** A schematic diagram illustrating the experimental design. The rats were divided into two groups, underwent coccygeal puncture (Co 5/6, Co 6/7) and injection of different drugs to perform RNA-Seq. PBS group: punctured IVD treated with PBS (*n* = 3), OKS@M-Exo group: punctured IVD treated with OKS@M-Exo (*n* = 3). **b** Volcano plot of differentially expressed genes (DEGs) at weeks 4 after treatment. **c** The Kyoto Encyclopedia of Genes and Genomes (KEGG) analysis results for the DEGs between OKS@M-Exo and PBS groups. Top twenty pathways were showed. The DEGs were filtered by Gene Ontology (GO) analysis in CC (**d**), BP (**e**) and MF (**f**). Top ten classifications of DEGs were showed in the graphic

To get insight into the molecular pathways of the protective effect of OKS@M-Exo on IVDD, we conducted transcriptomics RNA-seq analysis of IVD treated with or without OKS@M-Exo at 1 month after treatment (Fig. 7a). Differentially expressed genes (DEGs) were identified based on significant expression level differences (log_2_ FC > 1 and FDR < 0.05) between OKS@M-Exo and PBS groups. Transcriptome sequencing revealed distinct expression profiles in OKS@M-Exo group compared to PBS group, with 357 DEGs identified—215 upregulated and 142 downregulated genes (Fig. 7b and Fig. S22). Notably, genes related to ECM catabolic metabolism, such as *Mmp3*, *Mmp13*, *Adamts5* and senescence related genes, including *Tp53*, *Cdkn1a* and *Cdkn2a*, were significantly downregulated in OKS@M-Exo group. In contrast, ECM anabolic metabolism genes, *Col2a1* and *Acan*, were significantly upregulated in OKS@M-Exo group compared with PBS group. The Kyoto Encyclopedia of Genes and Genomes (KEGG) pathway enrichment analysis revealed significant enrichment of DEGs in OKS@M-Exo group within pathways such as “cell cycle”, “cell mitosis”, “p53 signaling pathway”, “cellular senescence” and “ECM-receptor interaction” (Fig. 7c). The “cell cycle” and “cell mitosis” are closely related to cell proliferation capacity. In addition, the “p53 signaling pathway” and “cellular senescence” are intricately linked to the aging process. The pathway “ECM-receptor interaction” is strongly correlated with the balance of ECM metabolism. Meanwhile, Gene Ontology (GO) enrichment analysis was used to classify and enrich for gene ontology functions of DEGs between OKS@M-Exo and PBS groups. Cellular component (CC) represents the location and organization of proteins in cells. Biological process (BP) denotes the biological activities in which genes and proteins are involved. Molecular function (MF) describes the activities and properties of protein molecules in biochemical reactions. The results of CC enrichment indicated that DEGs were associated with spindle components, encompassing spindle, spindle pore, spindle midzone, and microtubule, all of which played a role in cell mitosis (Fig. 7d). BP enrichment suggested that DEGs were mostly involved in cell proliferation processes, including cell division, chromosome segregation, mitotic spindle organization and cell cycle (Fig. 7e). Additionally, MF enrichment revealed that the DEGs were predominantly related to mitosis, including microtubule binding, ATP binding, and DNA replication origin binding (Fig. 7f). In summary, the KEGG and GO analysis results revealed that OKS@M-Exo could restore the proliferation ability and maintain the metabolic balance by reshaping the epigenetic information of aging NPCs.

## Discussion

IVDD is an intricate process intricately linked to aging-related diseases. As individuals age and disc degeneration progresses, senescent NP chondrocytes tend to increase or accumulate within the IVD, representing a pivotal factor in the development of disc degeneration.^3,44,45^ Major factors driving cellular senescence encompass genomic instability, telomere erosion, epigenetic modifications, disrupted proteostasis, and impaired autophagy.^7^ Recent study suggests that loss of epigenetic information speeds up the marks of aging, and notably, these changes can be reversed by epigenetic reprogramming.^17^ Therefore, the regulation of epigenetic information emerges as a promising avenue for restoring the normal state of aging NP Chondrocytes, opening up a new possibility for IVDD treatment.

Through genetic engineering and other methodologies, the modulation of epigenetic information has shown promise in reducing the accumulation of senescent cells. Researchers, such as Guan and Yang et al., have demonstrated that both genetic reprogramming and chemical reprogramming can effectively regulate epigenetic information, alter cell fate and present a potential strategy for delaying the senescence process.^17,46^ This is exemplified in studies on Hutchinson-Gilford progeria syndrome (HGPS), where senescent cell marks, including nuclear envelope abnormalities, telomere shortening, and oxidative stress, were reversed through over expression of OSKM.^18^ Chen et al. further observed that partial expression of OSKM in transgenic mice alleviated IVDD.^19^ To reset the epigenome without erasing cell identity, Lu et al. developed an in vivo overexpression vector. By ectopic expressing OSK, they facilitated axon regeneration following injury and reversed vision loss in a mouse model of glaucoma as well as in aged mice.^47^ In our study, we successfully constructed an overexpression OKS plasmid and demonstrated that OKS plasmid could reduce the characteristics of replicative senescence, including DNA damage, changes in histone methylation modification, low proliferation capacity, and the appearance of SASP. Simultaneously, after OKS plasmid treatment, the expression of *Mmp13* and *Adamts5* in aging NPCs decreased, while the expression of *Acan* and *Col2a1* increased, indicating that OKS plasmid could restore the metabolic balance of the aging NPCs (Fig. 1). However, an effective and non-harmful transfection method of OKS plasmid to aging NPCs remains scarce.

Exosomes, due to their inherent stability, low immunogenicity, biological compatibility^29^, and adaptability to diverse requirements^48,49^, have been widely acknowledged as a successful platform for drug delivery. Therefore, in our study, we utilized exosomes as carriers for OKS plasmids. In the course of our experiments, we observed a decrease in the ability of aging NPCs to internalize exosomes compared to their younger counterparts. Our results indicated that exosomes primarily entered NPCs through caveolae-mediated endocytosis (Fig. 2e–g). Therefore, through genetic engineering modifications, we enhanced the internalization capability of aging NPCs for exosomes by surface modification of exosomes with Cavin2 protein associated with caveolae endocytosis (Fig. 2l–n). This enhancement is considered a prerequisite and crucial condition for delaying the aging process of exosomes. Therefore, we used Cavin2 modified exosomes to load OKS plasmids (OKS@M-Exo).

Next, we investigated the efficacy of OKS@M-Exo in rejuvenating the epigenetics of aging NPCs. In vitro experiments using a replicative senescence model on NPCs demonstrated that OKS@M-Exo played a pivotal role in regulating epigenetic information, thereby restoring the normal function of aging NPCs (Fig. 3). Additionally, in vivo experiments corroborated the ability of OKS@M-Exo to delay IVDD, as illustrated in Fig. 4d. Like our previous studies and those of other scholars^50–54^, we used different segments of the tail of the same SD rat and divided them into different experimental groups, which can reduce the bias of the experimental results caused by individual differences in rats. Notably, M-Exo, in contrast, did not exhibit a similar positive effect, aligning with findings from other studies.^37,42^ We posit that this discrepancy may be attributed to the frequency of exosome injections affecting experimental outcomes: we administered a single injection, whereas other studies employed multiple injections. RNA sequencing results further revealed that OKS@M-Exo could inhibit senescence-related pathways, enhance the cell cycle, and restore proliferation capabilities (Fig. 7). Furthermore, OKS@M-Exo demonstrated a more pronounced alleviation of LBP compared to the PBS group through the evaluation of PATs and NGF expression (Fig. 6). These findings suggested that OKS@M-Exo could restore the normal function of aging NPCs by regulating their epigenetic information, thereby delaying IVDD and reducing the incidence of LBP.

In this study, we demonstrated that OKS@M-Exo is a promising candidate for alleviating IVDD and LBP by effectively addressing age-associated hallmarks and restoring youthful epigenetic information. It inhibits the senescence of NPCs and restores their proliferative capacity. Particularly, OKS@M-Exo exhibits significant potential for treating IVDD and LBP, offering an alternative for individuals facing the dilemma of choosing between conservative treatment and surgical procedures. The construction of OKS@M-Exo presents a promising strategy for ameliorating senescent cellular phenotypes in IVDD, with broad application prospects in tissue regeneration.

There are still some limitations in this study that need to be addressed. In both our research and previous studies, many scholars have constructed a 3-D cell culture system for further verification, which can encapsulate cells and form a cellular environment more similar to in vivo conditions compared with a 2-D culture system.^37,55^ In our future studies, we plan to use the 3-D culture systems for relevant in vitro experiments. Following RNA-Seq analysis, GO and KEGG analyses indicated that OKS@M-Exo could influence pathways related to senescence. In subsequent studies, we aim to further validate these specific pathways and identify which genes’ chromatin accessibility is affected using ATAC-seq and CUT-Tag methods.

## Materials and methods

### Isolation and culture of NP cells and BMSC cells

Adult male Sprague-Dawley (SD) rats weighing between 200 g and 250 g (6 to 8 weeks old) were used to isolate NPCs. And NP tissue from the rat tail vertebrae was collected. All procedures were approved by animal experimentation committee of Southern University of Science and Technology (SUSTech-JY202111008). As described previously,^40^ gelatinous NP tissue was isolated, cut into tissue fragments as small as possible. The fragments were enzymatically digested in trypsin (0.2%) for 30 min and collagenase II (0.25%) for 4 h. The tissue and cell suspensions were transferred to a new centrifuge tube, centrifuged at 1 000 r/min for 5 min, and the supernatant was discarded. The pellet was resuspended in complete Dulbecco’s Modified Eagle’s Medium (DMEM) medium containing 10% fetal bovine serum (FBS) (Invitrogen, USA) and 1% penicillin-streptomycin (Invitrogen, USA). For this study, the passage 2 NPCs were used as normal cells and passage 6 NPCs were used as senescent NPCs.

Adult male SD rats weighing between 200 g and 250 g (6 to 8 weeks old) were used to isolate BMSCs. And bilateral femur and tibia of rats were isolated and disinfected with 75% alcohol. We washed the bone with 0.9% sodium chloride solution, removed the two ends of bone with bone rongeur, exposed the bone marrow cavity, and flushed out the bone marrow with complete medium. The tissue and cell suspensions were transferred to a new centrifuge tube, centrifuged at 1 000 r/min for 5 min, and the supernatant was discarded. The pellet was resuspended in complete DMEM/F-12 containing 10% FBS and 1% penicillin-streptomycin. The passage 2 BMSCs were used for experiments.

### Construction and transfection of plasmid

Mammalian expression vector pcDNA3.1^+^ was selected to overexpress the proteins of interest. Recombinant vector encoding rat Oct4, Klf4, Sox2 and Cavin2 were constructed by Sangon Biotech company (Shanghai, CHN). According to the manufacturer’s protocol, the overexpressed vector was transfected into NPCs or BMSCs using Lipofectamine^TM^ 3000 (Invitrogen, USA). Cells transfected with overexpressed plasmids and then used for subsequent experiments by extracting RNA or proteins, etc.

### Immunofluorescence analysis

The NPCs were cultured in 24-well plates placed with cell coverslips for 48 h. After treated with OKS plasmids (with or without Lipofectamine 3000), M-Exo, and OKS@M-Exo, the cells were rinsed with PBS three times for 3 min each time, fixed with 300 μL 4% paraformaldehyde for 15 min, added with 200 μL 0.5% Triton X-100, permeated for 5 min and added 200 μL of 5% BSA to each well to block for 60 min. Dilute primary antibody was added into the cells and incubated at 4 °C overnight, then fluorescent secondary antibody was incubated at room temperature in the dark. 200 μL of Hoechst (Beyotime, CHN) was added to each well and incubated for 7 min at room temperature. After rinsing with PBS, images were acquired using a confocal laser scanning microscope (CLSM, ZEISS, GER).

### EdU incorporation

To detect the proliferation ability of cells, NPCs (3 × 10^5^ per well) were cultured in 24-well plates in a suitable medium with 10% FBS until reaching 50% confluency. The EdU assay was conducted following the manufacturer’s instructions using the EdU-488 detection kit (Beyotime, CHN). The nucleus was stained with Hoechst for 7 min. Then images were obtained under a fluorescence microscope (Leica, GER). The EdU^+^ cells were calculated by ImageJ 1.52n (National Institutes of Health, USA).

### Senescence-associated β-galactosidase (SA-β-Gal) staining

SA-β-Gal staining (Beyotime, CHN) was conducted following the manufacturer’s instructions. In brief, we completely immersed the cells in 1 mL of staining fixative and fixed at room temperature for 15 min. Then the cells were washed with PBS 3 times on the shaker for 5 min each time. Cells were incubated with staining working solution overnight in a 37 °C constant temperature drying oven, avoiding the influence of CO_2_. On the second day, we taken out the 6-well plate, removed the staining working solution, and rinsed with 70% ethanol to remove crystals and excess dye. Pictures were obtained under optical microscope (Leica, GER). Then we used ImageJ to calculate the number of β-Gal^+^ cells.

### Collection, purification and identification of exosomes

Exosomes were isolated by ultracentrifugation.^56^ Briefly, BMSCs (approximately 1 × 10^7^ cells) were cultured with exosome-free serum and culture medium, and the culture medium was collected. The collected medium was centrifuged at 800 g for 10 min in a 4 °C centrifuge. The supernatant was transferred to a new 50 mL centrifuge tube, centrifuged at 10 000 *g* for 30 min in a 4 °C centrifuge to further remove cells and cell debris. Then the supernatant was filtered through a 0.22 μm filter to remove particles larger than 200 nm. Exosomes pellet was obtained by ultracentrifugation at 120 000 *g* for 100 min in an ultracentrifuge (Beckman, USA). The exosome pellet was resuspended in a small volume of buffer for subsequent experiments or frozen in a −80 °C refrigerator for later use. The morphology of exosomes were captured by TEM. The number and size of exosomes were assessed by NTA. The specific markers on exosomes were detected by WB analysis.

### Exosomes labeling and internalization assay

The cellular uptake of exosomes by NPCs was measured by CLSM and FACS. For the CLSM analysis, purified exosomes were labeled with 4 μL PKH26 (Sigma-Aldrich, USA) according to the manufacturer’s instructions. Briefly, the diluted exosomes were added to the diluted PKH26 dye solution, incubated at room temperature for 5 min. Then the same volume of FBS was added to remove excess dye solution. After centrifugation with 100 kD ultrafiltration tube, the PKH26-labeled exosomes were obtained. The labeled exosomes were co-cultured with NPCs, and detect the internalization of exosomes in the cells by immunofluorescence and flow cytometry. Cytoskeleton was stained with phalloidin (Beyotime, CHN) and nucleus was labeled with Hoechst. Images were acquired by CLSM and analyzed with ImageJ. Similarly, the labeled exosomes were co-cultured with cells for 6 h. And the cells were digested with trypsin, centrifuged, resuspended with PBS and transferred to the flow cytometer tube. We detected them using a flow cytometer (BD Biosciences, USA) and analyze the data with FlowJo X software (Tree Star, USA).

### Lysosome escape

The lysosome escape of exosomes was evaluated. NPCs were seeded either in 24-well plates (3 × 10^5^/well) and incubated for 12 h prior to the following experiments. PKH26 labeled exosomes were added to the FBS-free DMEM medium and cells were cultured for 1, 2 or 9 h. Cells were washed by PBS thrice and stained with a Lysotracker (Thermo, USA) for 15 min and Hoechst for the nucleus for 5 min. The images were taken by using a CLSM.

### DNA loading into exosomes by electroporation

To obtain the exosomes loaded with OKS plasmids, we re-suspended exosomes using electroporation buffer,^57^ transferred them into an electroporation cup, and added OKS plasmid at a ratio of 10 μg exosomes (approximately 3 × 10^8^ particles as measured by NTA) to 5 μg plasmid, making a final volume of 50 μL. We placed the electroporation cup into an electroporator and set the conditions to 400 V and 125 μF for two pulses. After electroporation, exosomes were transferred from the electroporation cup to a 1.5 mL tube. And 1 mmol/L EDTA solution was added to reduce aggregation of plasmid DNA.^58^ Then the exosome suspension was transferred into an ultrafiltration tube and centrifuged at 5 000 r/min for 10 min at 4 °C to remove excess buffer and unincorporated OKS plasmid. After electroporation, the exosomes were re-suspended for subsequent experiments.

### Patient samples

We obtained human NP tissue specimens from patients hospitalized in the Department of Spine Surgery, The First Affiliated Hospital of Sun Yat-sen University. Patients were informed about the purpose of collecting clinical specimens and signed consent forms. This method was approved by the ethics committee (No: [2020] 488). We divided the acquired NP tissue into two groups based on the Pfirrmann grading method from MRI images: mild degeneration (grade I-II) and severe degeneration (grade III-V). Patients with a history of spinal infections, tumors, or previous surgeries were excluded from the study. According to the standard operating procedures of each surgery, NP tissue was extracted under sterile conditions and placed in 4% paraformaldehyde solution and stored in a 4 °C refrigerator.

### Animal model and surgical technique

The animal experimental protocol was approved by the Ethics Committee of Southern University of Science and Technology (SUSTech-JY202111008). Adult male SD rats weighing between 200 g and 250 g (6 to 8 weeks old) were housed under SPF conditions. We used different segments on the tail of the same SD rat and divided it into different experimental groups, which can reduce the bias caused by individual variation between rats on the experimental results. In each group, 5 rats were sacrificed for IHC, HE staining, MRI and Micro-CT at each experimental time-point. All surgeries performed were similar to the protocol described in the other studies^39–41^. In brief, after adaptive feeding for a period of time, the operation and different treatment groups could be done. The rats were anesthetized with 2% (w/v) sodium pentobarbital (40 mg/kg), and the experimental disc level (Co 5/6, Co 6/7, Co 7/8, Co 8/9 and Co 9/10) in the coccygeal vertebrae of each rat was located by digital palpation and confirmed by trial radiography. After skin sterilization, a syringe needle (27-G) was used to puncture the AF layer (~4 mm in depth from the skin) along the vertical direction. And the needle was rotated 360° and leaving it in place for 30 s to induce IVDD. Two weeks later, 2 μL PBS, 2 μL OKS plasmid (50 μg/mL), 2 μL M-Exo or OKS@M-Exo (100 μg/mL) were injected into the IVD of the rats, respectively. To minimize secondary damage from drug administration, the drugs were injected through the same needle tract into the IVD. Four or eight weeks after the second surgery, the rats were anesthetized for imaging studies and euthanized afterward for histological examination of tissue samples.

For the behavioral test, we used the anterior lumbar puncture model of rats. In brief, the rats were intraperitoneal injected with 2% (w/v) sodium pentobarbital (40 mg/kg), and the experimental disc level (L4/5 or L5/6) was located by bilateral iliac ridge line, which is directly opposite to the L4/5 IVD. After skin sterilization, the internal organs of the rats were removed to the right side to expose the L4/5 IVD, and a syringe needle (27-G) was used to puncture the AF layer (controlled to a depth of 1.5 mm using fine-tipped forceps) along the vertical direction. The syringe needle was rotated in the axial direction by 360° and held in place for 30 s to induce IVDD. Two weeks later, 2 μL PBS, 2 μL OKS@M-Exo (100 μg/mL) were injected into the IVD of the rats, respectively. The pain behavior of the rats was examined for 6 weeks.

### MRI processing and analysis

Rats from different groups were examined with MRI. The radiological examination of rats was conducted 4 and 8 weeks postoperatively. After intraperitoneal injection of 2% (w/v) sodium pentobarbital (40 mg/kg), the rats were placed in the MRI rat coil (1.5 T vExpMR, GSMED, CHN). We adjusted the parameters (thickness: 3 mm, field strength: 1.5 T, repetition time: 2 400, echo time: 110, weight image: T2WI), and acquired sagittal and cross-sectional images of the rat’s lumbar or tail spine. The grayscale of the IVD in MRI was determine d using ImageJ software to reflect the water content of the disc. Meanwhile, we performed Pfirrmann grading to explore the degree of disc degeneration in different groups by three independent researchers and performed statistical analysis.

### Micro-CT

The rats were anaesthetized and then sacrificed. Tails of rats were isolated and fixed in 4% paraformaldebyde. A Micro-CT imaging system (SkyScan126, Bruker) was used to scan the tail tissue. We reconstructed it to get a sagittal picture, the height of the vertebra and disc in CT was determined using ImageJ software and we then calculated the DHI to evaluate the degree of IVDD.

### Histological analysis

Tissue samples of IVD were collected at weeks 4 and 8 postoperatively. All samples were fixed in 4% paraformaldehyde for 48 h and decalcified in EDTA decalcification fluid for 4 weeks. Then the samples were rinsed overnight in distilled deionized water, dehydrated in gradient alcohol, clear in xylene, and embedded in paraffin. After embedding, the wax blocks were frozen overnight in −20 °C. Serial sections of 5 μm thickness were performed along the sagittal plane of the specimen using a tissue slicer (Leica, GER). Sections were baked overnight at 60 °C. The structural changes of the IVD were investigated by H&E staining. The collagen remodeling was investigated by Safranin O staining. In this study, the histological grades (Table S2) of rat IVD were determined according to the previous studies^59,60^. The expression of MMP13, ACAN and P21 were determined by IHC and performed statistical analysis.

### Behavioral studies

#### Pressure algometry

An algometer (Ugo Basile) was used to measure the sensitivity of deep axial lumbar tissue to noxious mechanical force, as previously described by Kim et al.^61^ Simply, the force threshold was measured by pressing a 0.5 cm^2^ device tip directly on the dorsal skin over the IVD (L4/L5). The force was applied at a rate of approximately 100 g/s until the rat vocalizes or exhibits obvious avoidance behavior. Each rat was tested three times, with at least a 5-min interval between each measurement. And the instrument was set to a 1 000 g upper limit to prevent injury to the rats.

#### Von Frey test

We tested the sensitivity to innocuous mechanical stimuli by measuring the paw withdrawal response following von Frey filament (Stoelting, Wood Dale, IL) stimulation. The rats were placed in transparent plastic enclosures with a metal wire mesh floor, allowing the von Frey filaments to easily access the rats’ hind paws during testing. The test started at 4.31 of moderate intensity. If there was no response, the intensity of primary stimulation was increased. If foot retraction reflex occurs, reduce primary stimulation. Each rat was examined 3 times, at least 5 min apart, and the measurements were recorded.

#### Hargreaves test

The response of the skin on the heel of the large hind limb to thermal injurious stimuli was measured by a thermal flat plate tester (Plantar Test 7370, Ugo Basile, ITA). During the test, infrared thermal stimulation was produced by a movable heat radiation light source through a transparent glass plate and focused on the heel of the rat’s hind limb. The intensity of thermal stimulation is appropriate for the withdrawal reaction of normal rats under 15–20 s light, and the incubation period of thermal stimulation of rats can be accurately recorded (accuracy up to 0.1 s). The bilateral posterior foot was measured 3 times at a time interval of 5 min, and the mean of the 3 withdrawal latency periods was used for the final statistical analysis

### RNA-seq

The RNA-seq procedures were conducted according to the previous studies^37,54^. Briefly, total RNA was extracted using the RNA extraction kit and stored at −80 °C. Then, a VAHTS Stranded RNA-seq Library preparation kit for Illumina (YEASEN; CHN) was used to construct strand-specific libraries. HISAT2 was used to compare the valid data of the sample to the reference genome for statistical Mapping information. Gene counts were obtained using HTseq, and gene expression was determined using the RPKM technique. The DESeq2 (1.12.4) algorithm was used to filter the differentially expressed genes after significance and FDR analysis, and the criteria were as follows: (i) log_2_ FC > 1 and (ii) FDR < 0.05. GO and KEGG analysis was performed using DAVID (https://david.ncifcrf.gov/, Version 6.8).

### Statistical analysis

Statistical analysis was performed with GraphPad Prism 9.0 software (La Jolla, CA, USA). Data are shown as mean ± SD of triplicates unless otherwise indicated. Statistical analysis was performed using a two-tailed Student’s *t*-test or one-way ANOVA with Tukey’s multiple comparisons, as described. A *P* value less than 0.05 was designated as statistically significant.

## Supplementary information

Supplementary Information

## Data Availability

All data associated with this study are present in the paper.
