# Supplementary material for: Reprogramming to restore youthful epigenetics of senescent nucleus pulposus cells for mitigating intervertebral disc degeneration and alleviating low back pain
Source: Bone Res. 2025 Mar 12;13:35. doi: 10.1038/s41413-025-00416-1 (PMC11903667; doi:10.1038/s41413-025-00416-1)
Supplement: Supplementary file 1 — Supplementary Information [file 41413_2025_416_MOESM1_ESM.pdf]

Supplementary information for:

Reprogramming to Restore Youthful Epigenetics of Senescent Nucleus Pulposus Cells for Mitigating Intervertebral Disc Degeneration and Alleviating Low Back Pain

Wenzheng Ma[1, 2, 3†], Wantao Wang[1, 2, 3†], Lei Zhao[3], Jinghao Fan[3], Lei Liu[3], Lin Huang[3], Baogan Peng[4], Jianru Wang[1, 2], Baoshan Xu[5], Hongmei Liu[3, *], Decheng Wu[3, *], Zhaomin Zheng[1, 2, *]

*Corresponding authors: Hongmei Liu, Decheng Wu, Zhaomin Zheng
Email: liuhm@sustech.edu.cn (H.L.); wudc@sustech.edu.cn (D.W.); zhzhaom@mail.sysu.edu.cn (Z.Z.)

†These authors contributed equally to this work.

This file includes:
Supporting Information of Materials and methods
Figures and legends S1 to S21
Table S1 and S2

Supporting Information of Materials and methods

Real-time quantitative reverse transcription PCR (RT-qPCR)

Total RNA was extracted using the RNA extraction kit. The complementary DNA was reverse transcribed using ReverTra Ace qPCR RT Kit. The reaction was performed on a MasterCycler (Thermo Fisher Scientific, USA). RT-qPCR was performed in a Quant Studio 5 Flex System (Thermo Fisher Scientific, USA) with SYBR qPCR SuperMix Plus (novoprotein, CHN). The total volume of each PCR reaction mixture was 20 μL, which included 10 μL of SYBR qPCR SuperMix Plus, 0.6 μL of each primer (10 μmol L^{-1}), 0.4 μL of cDNA template (50 μg mL^{-1}), 0.4 μL of ROXII and 8 μL of RNase Free Water. The RT-qPCR reactions conditions were: 95 °C for 1 min; 95 °C for 20 s, 60 °C for 1 min and for 40 cycles. Expression values were normalized to β-actin or GAPDH, and the data were presented as fold-change using the equation $2^{-\Delta\Delta CT}$, as recommended by the manufacturer. The sequences of the primers used are listed in Table S1. All the experiments were performed on three separate occasions.

Western blotting

Treated cells were isolated using radioimmunoprecipitation assay (RIPA) lysis buffer (Beyotime, CHN) on ice and centrifuged at 12,000 g for 20 min at 4 °C, and the supernatants were collected. The protein concentrations were measured using a bicinchoninic acid protein assay kit and an xMark microplate absorbance spectrophotometer. Equal amount of protein was separated on sodium dodecyl sulfate-polyacrylamide gel electrophoresis (SDS-PAGE) and transferred to polyvinylidene difluoride (PVDF) membranes (Millipore, USA). After blocking in 1 × TBST with 5% bovine serum albumin (BSA), the membranes were incubated with specific primary antibodies overnight at 4 °C. Subsequently, the membranes were incubated with the corresponding secondary antibodies for 1 h at room temperature and washed again prior to signal detection with a ChemiDoc Touch Gel Imaging System and Image Lab Touch software (Bio-Rad, USA). Antibodies used in this study were MMP13 (abcam, 1:3000), ADAMTs5 (abcam, 1:1000), TNF-α (abcam, 1:1000), IL-1β (abcam, 1:1000), Cavin2

(proteintech, 1:1000), CD9 (Proteintech, 1:1000), CD63 (Proteintech, 1:1000), CD81 (Proteintech, 1:1000), β-actin (abcam, 1:5000). Density of bands in three independent experiments was quantified by ImageJ 1.52n (National Institutes of Health, USA), and the protein levels were normalized to β-actin.

Osteogenesis and lipid differentiation of BMSCs

The BMSCs were seeded into 6-well plates. The osteogenesis differentiation of BMSCs was induced by DMEM/F-12 medium with 10% FBS, 1% double antibiotics, 1% glutamine, 1% β-glycerophosphate, 0.2% ascorbate acid and 1 μM dexamethasone. The medium was changed every three days. Three weeks later, the cells were stained with alizarin red. Lipid differentiation was induced by DMEM/F-12 medium with 10% FBS, 1% double antibiotics, 1% glutamine, 2 μM insulin, 1 μM 3-isobutyl-1-methylxanthine, 1 μM rosiglitazone and 1 μM dexamethasone for three days. Then, cells were cultured with complete medium containing 1% glutamine and 2 μM insulin for one day. The entire process lasted 20 days and the cells were stained with oil red O.

Identification of BMSCs surface antigen by flow cytometry

BMSCs were digested with trypsin and resuspended in DMEM/F-12 medium containing 10% FBS and 1% double antibiotics. The cells were washed with phosphate-buffered saline (PBS) thrice. Subsequently, the cells were incubated with anti-HLA-DR-APC, anti-CD19-V610, anti-CD11b-APC, anti-CD73-FITC, anti-CD90-APC and anti-CD105-PE antibodies, respectively, for 30 min. The cell suspension was then centrifuged at 1000 rpm for 5 min. Finally, the cell suspension was transferred into a new detection tube, followed by the detection of cell surface antigen using flow cytometry (BD Biosciences, USA).

Dual luciferase reporter assay

The reporter vector containing the octamer motif and the firefly luciferase reporter gene was purchased from GeneChem. The reporter vector was co-transfected with the internal control pRL-TK vector (Beyotime, CHN) in NPCs using Lipofectamine 3000. After 48 h, NPCs were treated with OKS plasmids, M-Exo, or OKS@M-Exo for 24 h and luciferase activity was assessed using the Luciferase Reporter Assay System according to the manufacturer's instructions (Beyotime, CHN).

Supporting Figures and legends

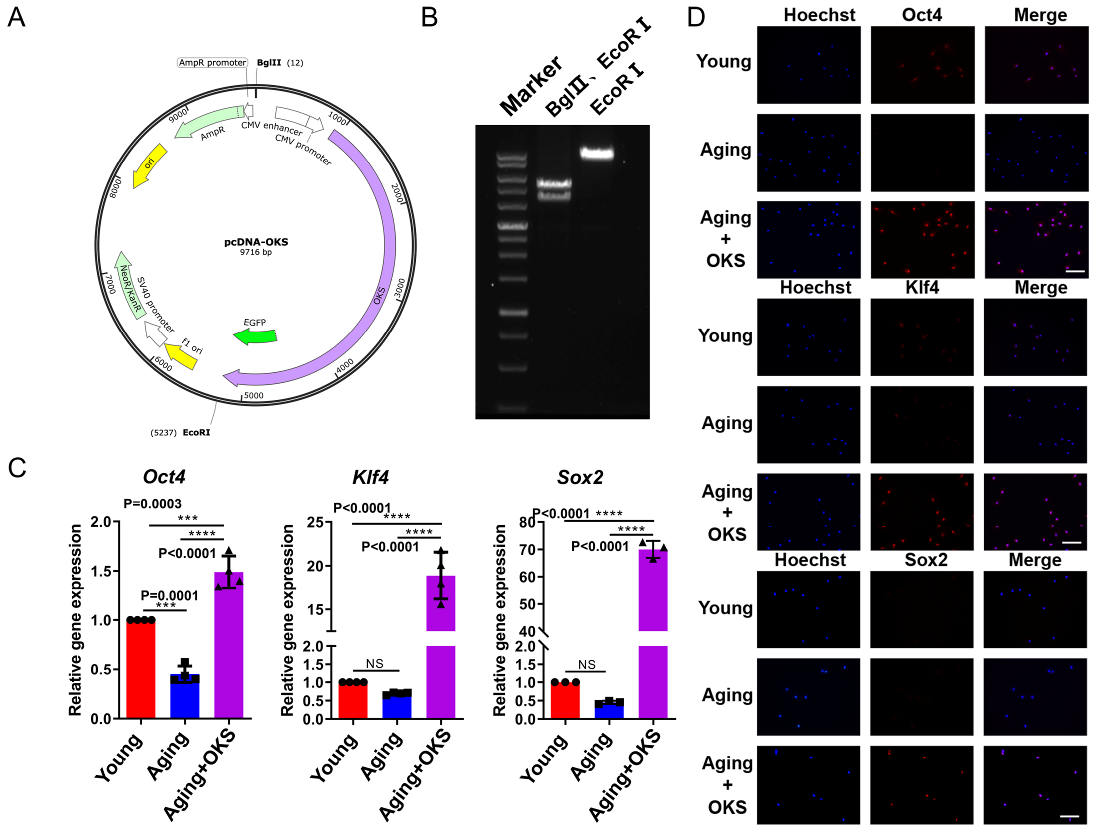

Fig. S1. The OKS plasmid was successfully constructed and expressed in the NPCs. (A) Plasmid map of the OKS overexpression plasmid. (B) Agarose Gel Electrophoresis assay showing two fragments of OKS plasmid cut by restriction endonuclease. (C) RT-qPCR analysis of expression level of *Oct4*, *Klf4* and *Sox2* in Young group (passage 2 NPCs), Aging group (replicative senescence model, passage 6 NPCs) and the Aging+OKS group (passage 6 NPCs treated with OKS plasmids). (n = 4/group). (D) Representative confocal immunofluorescence micrographs showing the expression of Oct4, Klf4 and Sox2 (red) in NPCs. Scale bar, 100 μm. ***$P < 0.001$, ****$P < 0.0001$, NS means none sense, one-way ANOVA with Tukey's multiple comparisons.

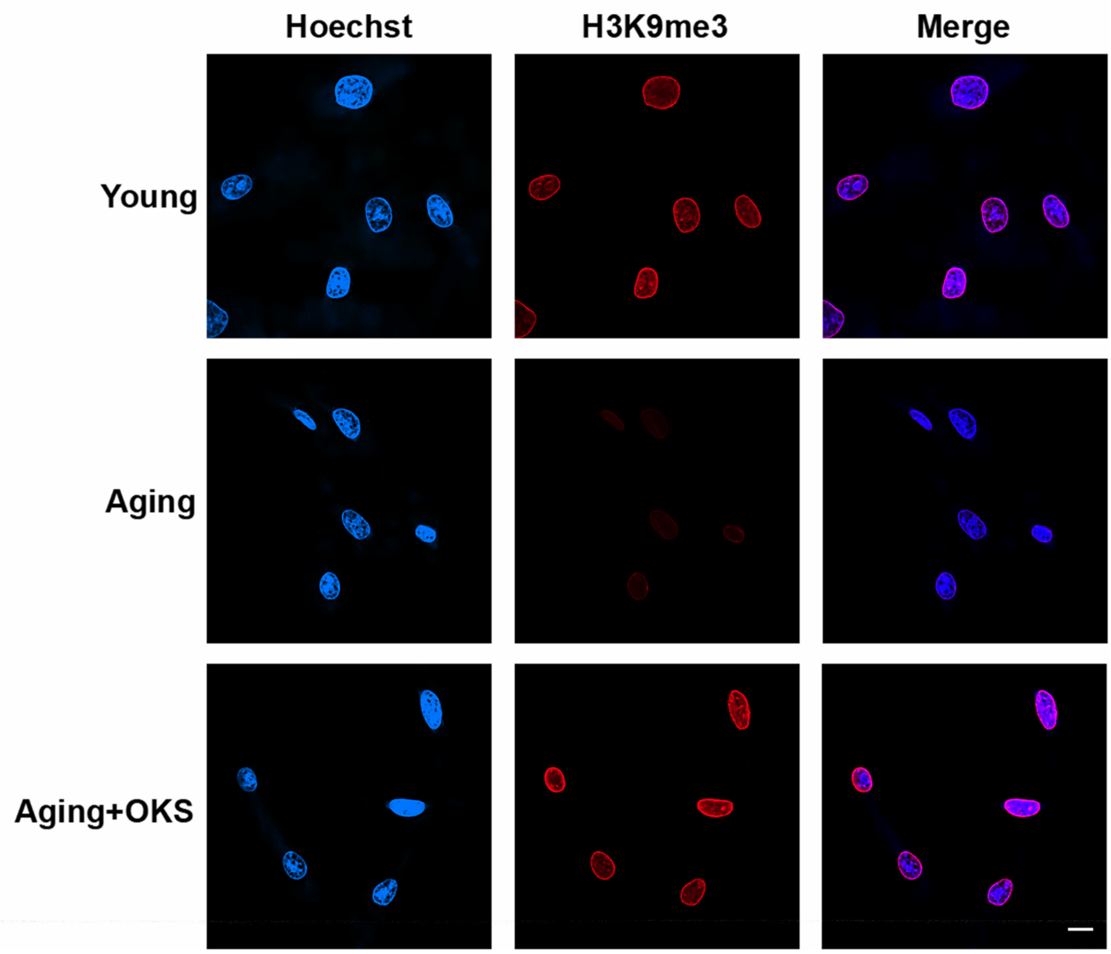

Fig. S2. OKS could increase the expression of H3K9me3 in senescent nucleus pulposus cells. Representative confocal immunofluorescence micrographs showing the expression of H3K9me3 (red) in Young group (passage 2 NPCs), Aging group (replicative senescence model, passage 6 NPCs) and the Aging+OKS group (passage 6 NPCs treated with OKS plasmids). Scale bar, 20 µm.

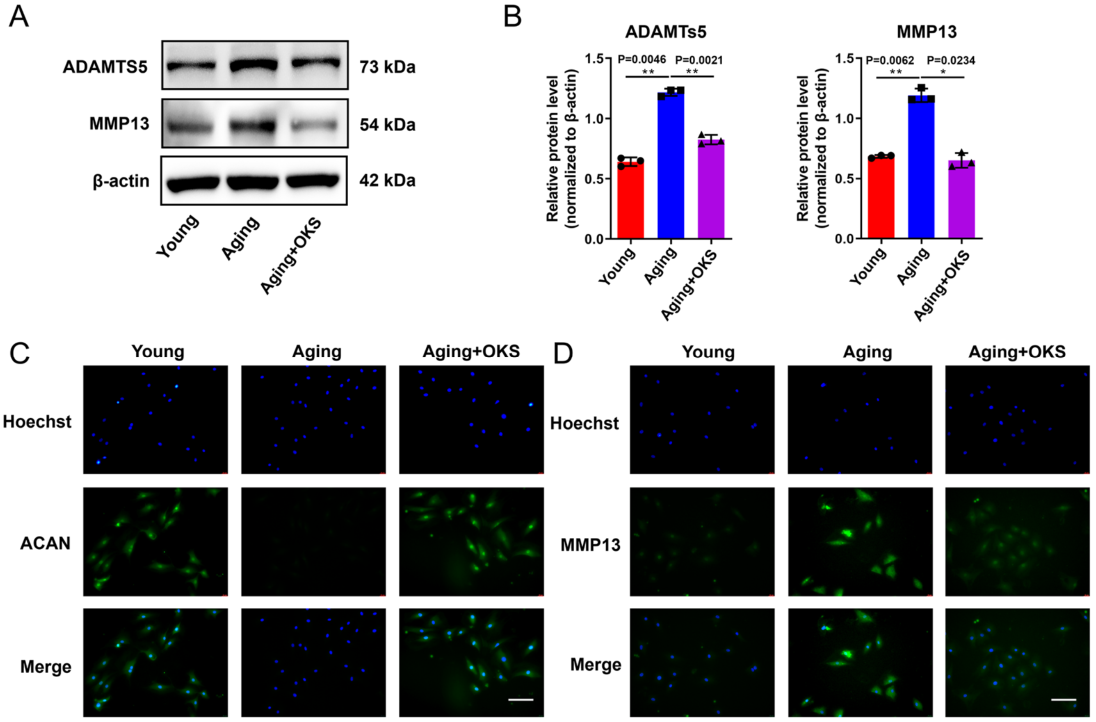

Fig. S3. Partial expression of OKS could restore the anabolic balance. (A,B) WB analysis (A) and semi-quantification (B) of ADAMTs5 and MMP13 in Young group (passage 2 NPCs), Aging group (replicative senescence model, passage 6 NPCs) and the Aging+OKS group (passage 6 NPCs treated with OKS plasmids). (n = 3/group). (C,D) Representative confocal immunofluorescence micrographs showing the expression of ACAN (C) and MMP13 (D). Scale bar, 100 μm. *P < 0.05, **P < 0.01, one-way ANOVA with Tukey's multiple comparisons.

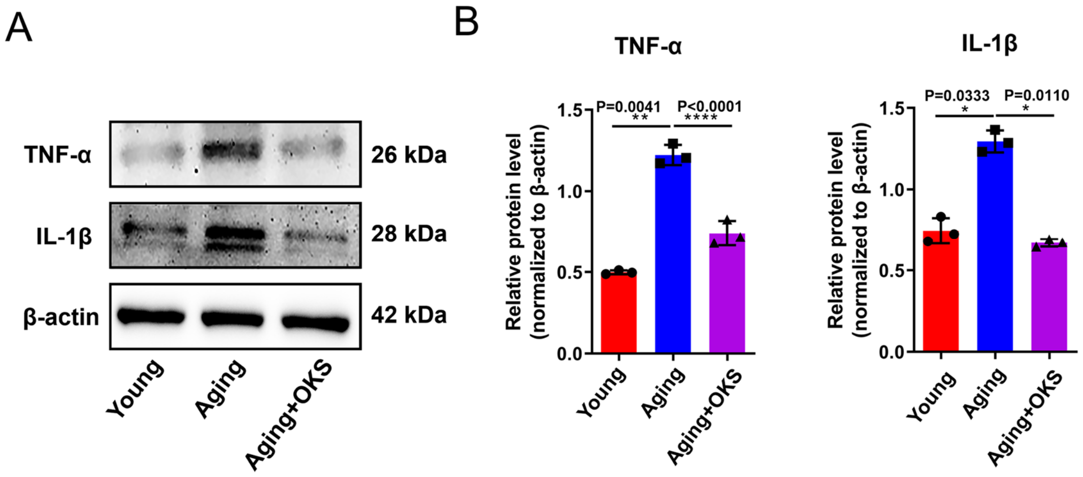

Fig. S4. Partial expression of OKS could downregulate the expression of inflammatory factor. (A,B) WB analysis (A) and semi-quantification (B) of TNF-α and IL-1β in Young group (passage 2 NPCs), Aging group (passage 6 NPCs) and the Aging+OKS group (passage 6 NPCs treated with OKS plasmids). (n = 3/group). *$P < 0.05$, **$P < 0.01$, ****$P < 0.0001$, one-way ANOVA with Tukey's multiple comparisons.

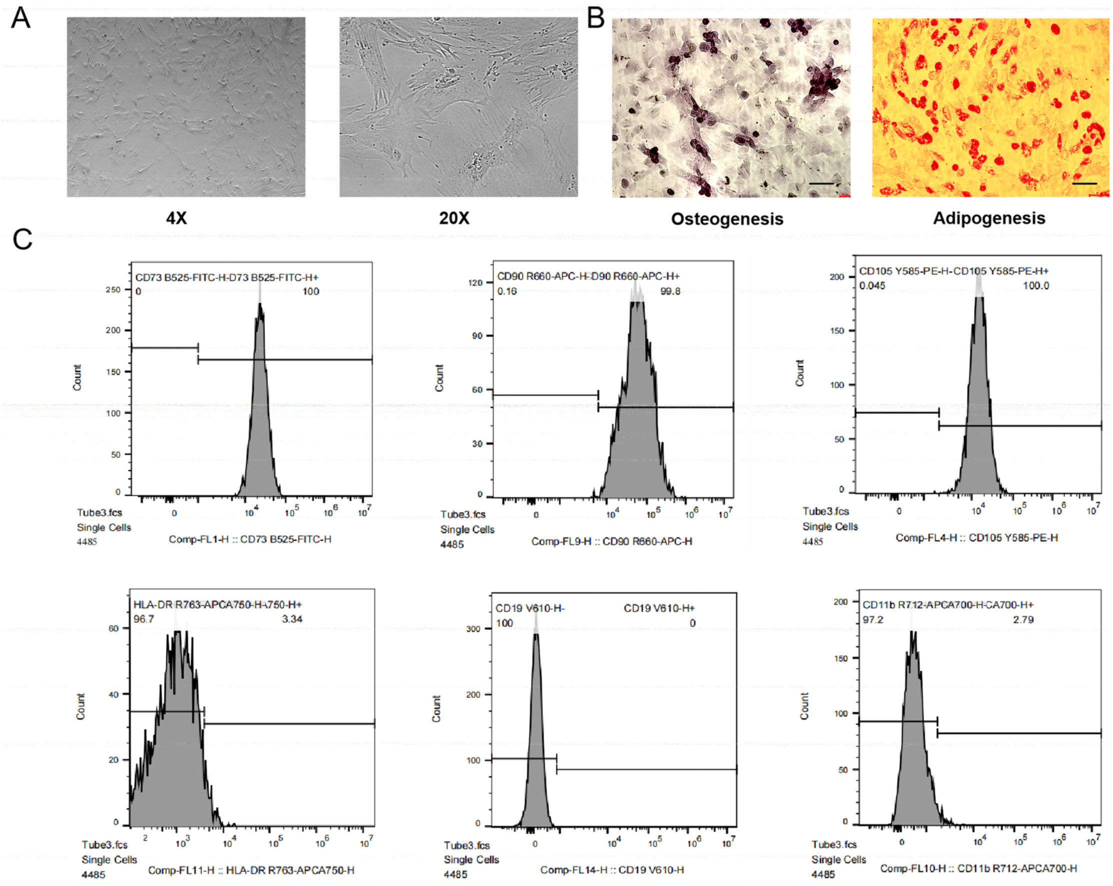

Fig. S5. Characterization of BMSCs. (A) Light microscopy images of BMSCs, displayed spindle-like morphology. (B) Alizarin red staining and Oil red staining suggested BMSCs were able to differentiate to osteocyte (Left) and adipocyte (Right). Scale bar, 50 μm. (C) Surface markers of BMMSCs analyzed by flow cytometry. BMSCs were positive for CD73, CD90 and CD105, and negative for HLA-DR, CD19 and CD11b.

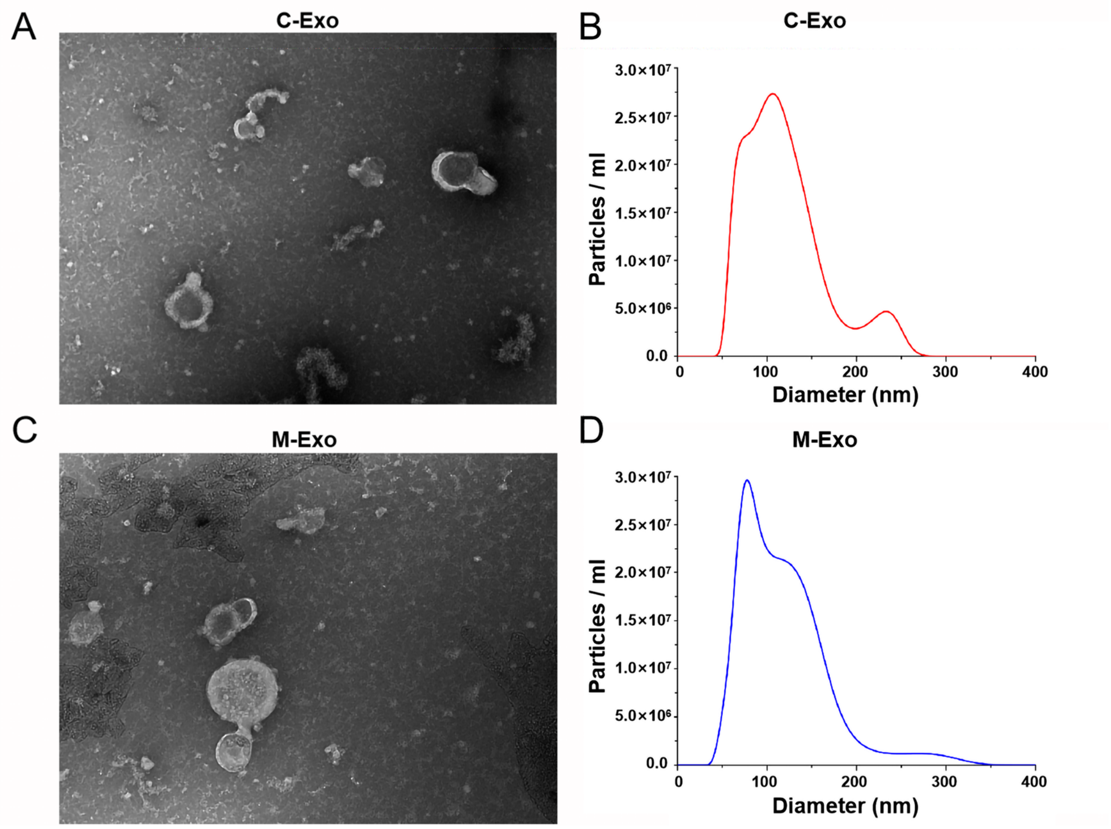

Fig. S6. The morphology and size of C-Exo and M-Exo. (A) Representative images of TEM of C-Exo (from normal BMSCs). Scale bar, 100 nm. (B) NTA results of C-Exo. (C) Representative images of TEM of M-Exo (from Cavin2-engineered BMSCs). Scale bar, 100 nm. (D) NTA results of M-Exo (from Cavin2-engineered BMSCs).

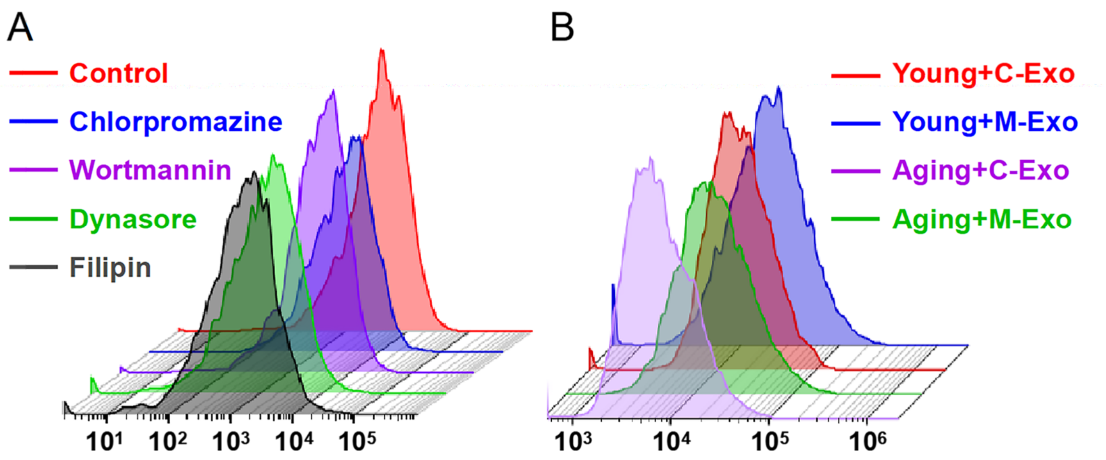

Fig. S7. Cavin2 enhanced the Caveolae-dependent endocytosis of exosomes in aging NPCs. (A) Fluorescence intensity of PKH26-labeled C-Exo taken up by NPCs under different inhibition conditions by flow cytometry. Chlorpromazine: clathrin-mediated endocytosis inhibitor; Wortmannin: inhibitor of the phosphoinositide 3-kinase; Dynasore: dynamin inhibitor; Filipin: Caveolae-dependent endocytosis inhibitor. (B) Fluorescence intensity of PKH26-labeled C-Exo or M-Exo taken up by young or aging NPCs by flow cytometry.

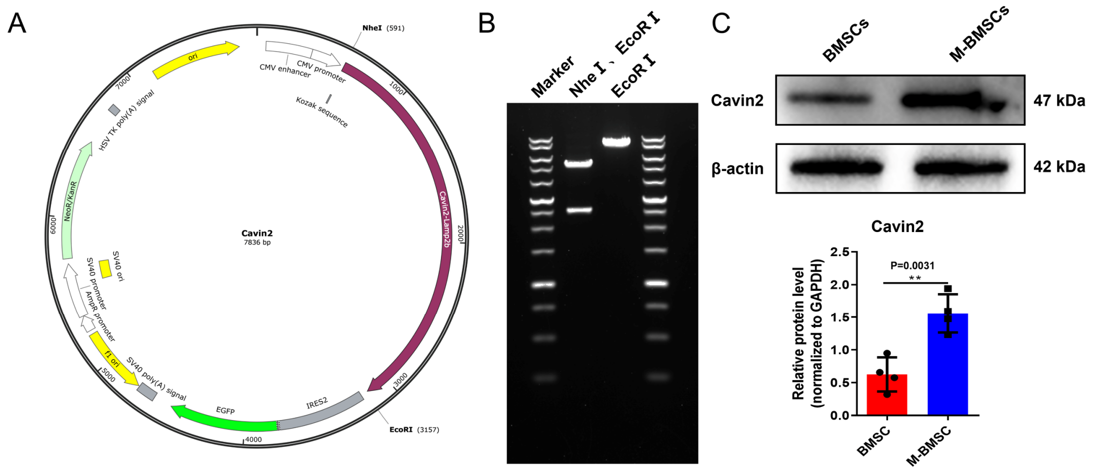

Fig. S8. The Cavin2 plasmid was successfully constructed. (A) Plasmid map of the OKS overexpression plasmid. (B) Agarose Gel Electrophoresis assay showing two fragments of Cavin2 plasmid cut by restriction endonuclease. (C) WB analysis and quantification of Cavin2 and β-actin in BMSCs transfected with Cavin2 plasmids. β-actin served as the loading control. (n = 4/group). **P < 0.01, two-tailed Student's *t*-test.

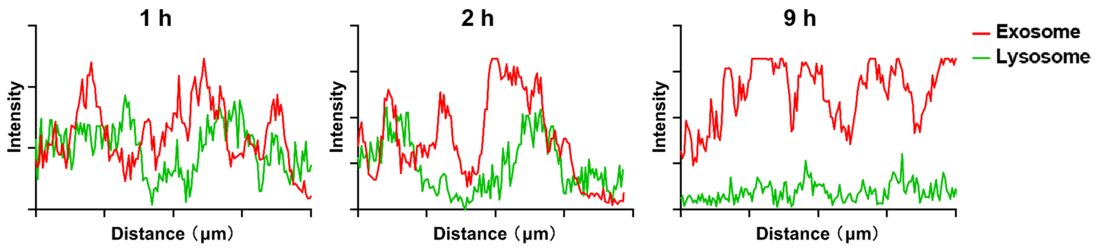

Fig. S9. The exosomes effectively escaped from the lysosomes. The confocal immunofluorescence intensity of exosome (red) and lysosome (green) in NPCs co-cultured with exosomes for 1 h, 2 h and 9 h.

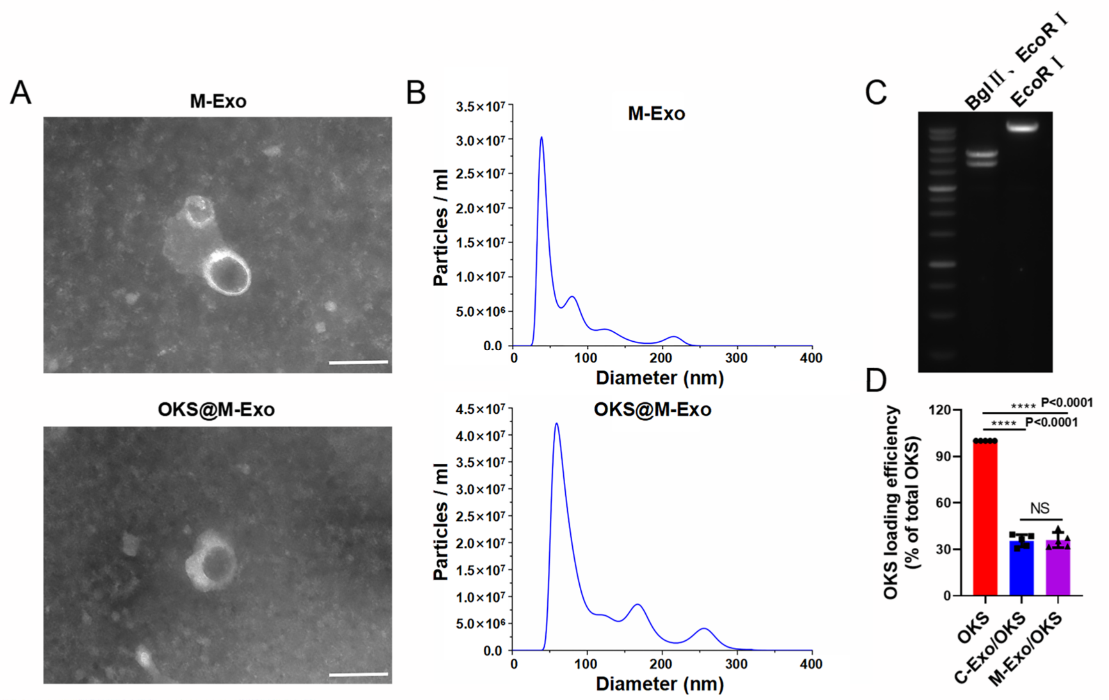

Fig. S10. Electroporation did not affect the morphology and size of exosomes, and the structure of OKS plasmid. (A) Representative images of TEM of M-Exo (from Cavin2-engineered BMSCs) and OKS@M-Exo (OKS plasmid electroporated into M-Exo). Scale bar, 100 nm. (B) NTA results of M-Exo (from Cavin2-engineered BMSCs) and OKS@M-Exo (OKS plasmid electroporated into M-Exo). (C) Agarose Gel Electrophoresis assay showing two fragments of OKS plasmid, extracted from OKS@M-Exo, cut by restriction endonuclease. (D) Quantification of the loading efficiency of different exosome preparations. (n = 5/group). ****$P < 0.0001$, NS means none sense, one-way ANOVA with Tukey's multiple comparisons.

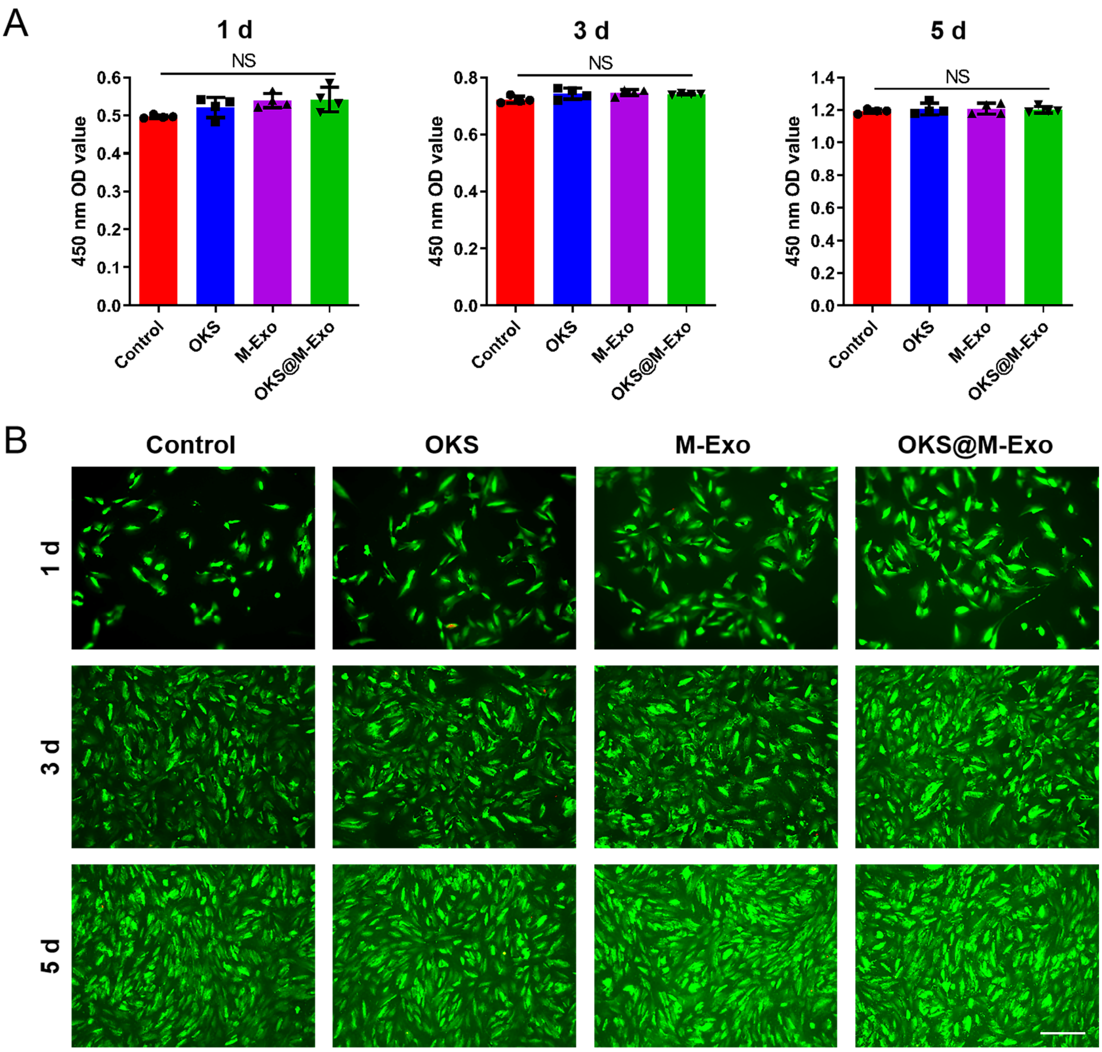

Fig. S11. OKS@M-Exo has no toxic and side effects on normal NPCs. (A,B) Cell counting kit-8 (CCK-8) assay (A) and live/dead staining (B) of the NPCs. The Young NPCs were used as the Control group and treated with OKS plasmid (OKS group), M-Exo (M-Exo group) and OKS@M-Exo (OKS@M-Exo group). (NS means none sense, one-way ANOVA with Tukey's multiple comparisons, n = 4/group). Scale bar, 200 μm. NS means none sense, one-way ANOVA with Tukey's multiple comparisons.

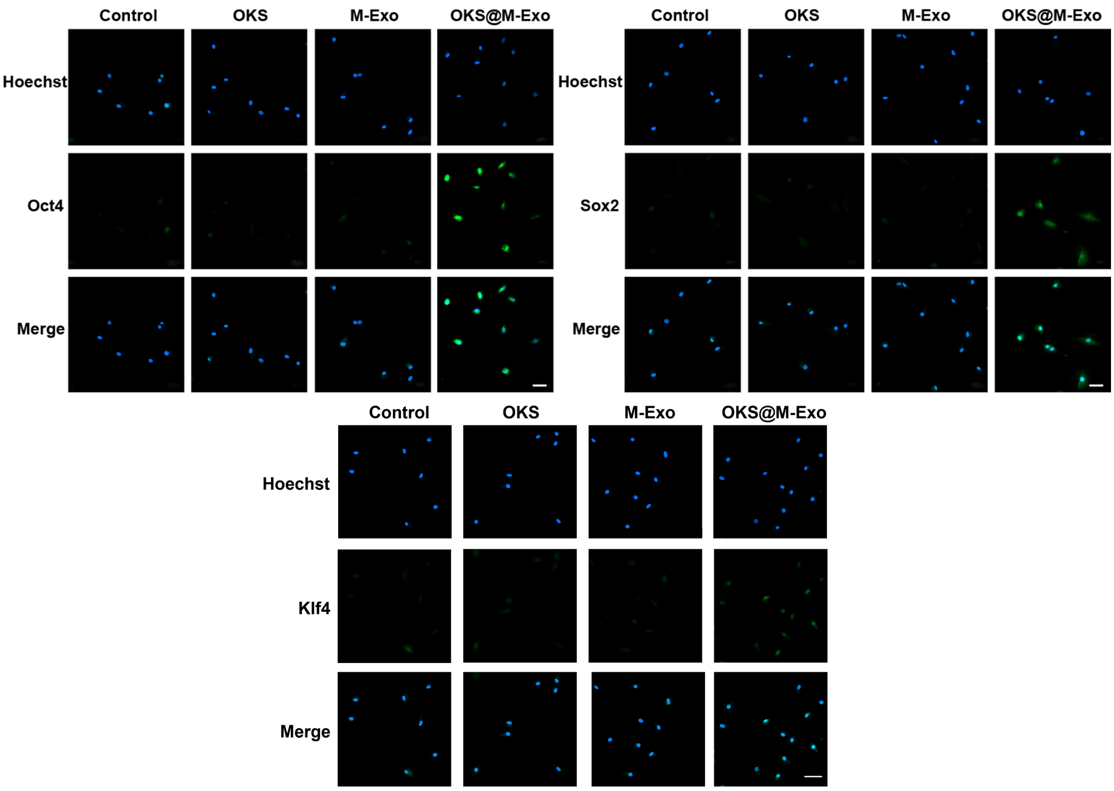

Fig. S12. Aging NPCs treated with OKS@M-Exo resulted in the high expression of OKS. Representative confocal fluorescence micrographs showing the expression of Oct4, Sox2, and Klf4 (green) in aging NPCs treated with Control, OKS, M-Exo and OKS@M-Exo groups. Scale bar, 50 μm.

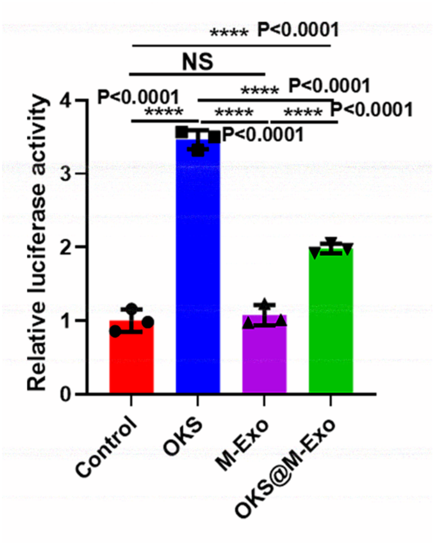

Fig. S13. OKS plasmids delivered into NPCs via OKS@m-exo have transcriptional function. Relative luciferase activity in aging NPCs treated with Control, OKS (with Lipofectamine 3000), M-Exo, and OKS@M-Exo groups. (n = 3/group). ****P < 0.0001, NS means none sense, one-way ANOVA with Tukey's multiple comparisons.

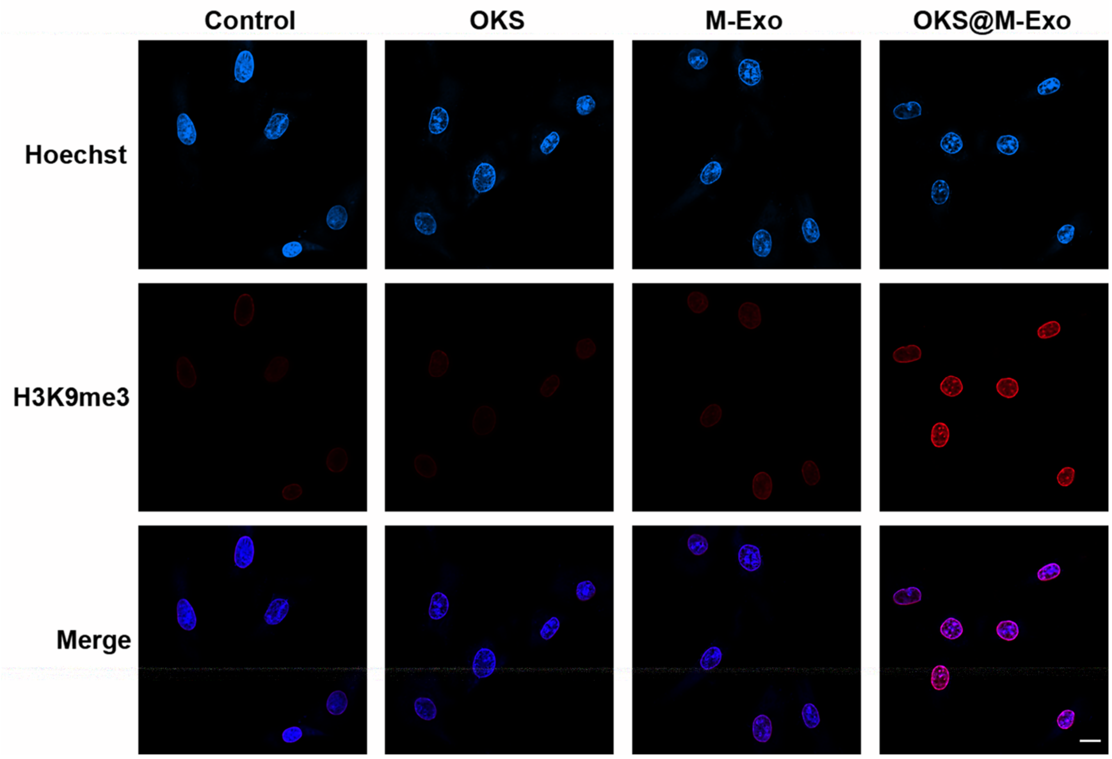

Fig. S14. Aging NPCs treated with OKS@M-Exo resulted in the high expression of H3K9me3. Representative confocal fluorescence micrographs showing the expression of H3K9me3 (red) in aging NPCs treated with Control, OKS, M-Exo and OKS@M-Exo groups. Scale bar, 20 μm.

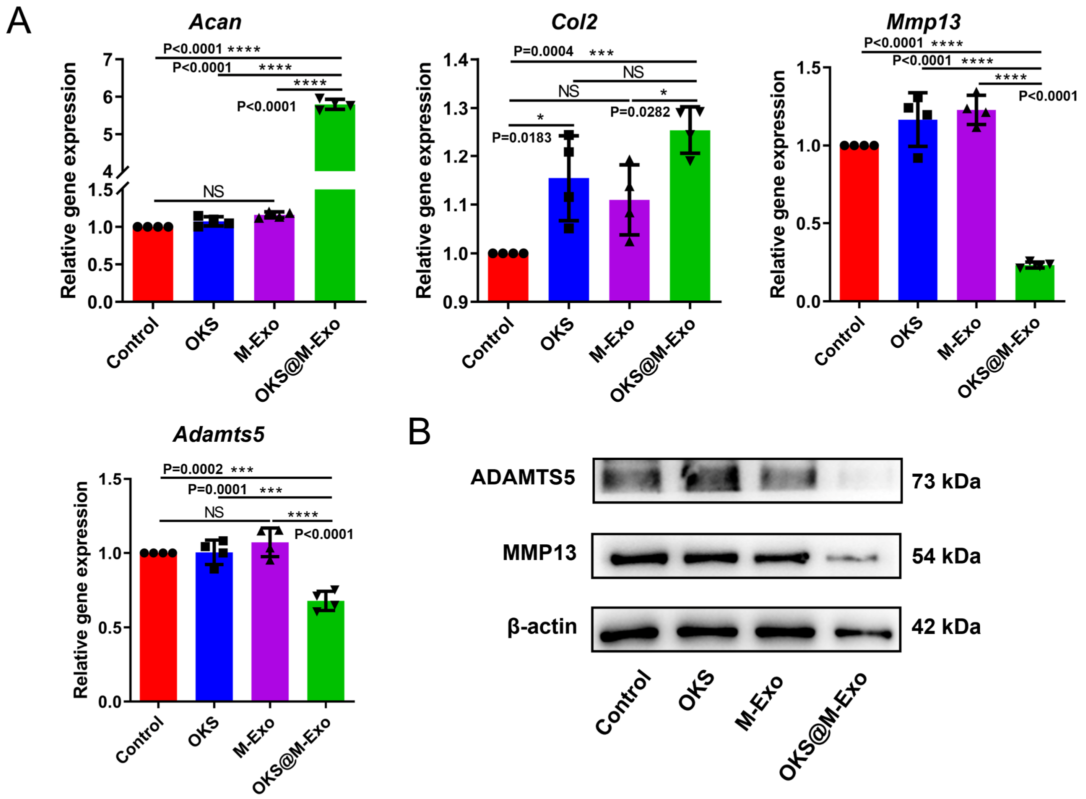

Fig. S15. Aging NPCs treated with OKS@M-Exo could restore the anabolic balance. (A) RT-qPCR analysis of senescence-associated anabolism factors *Acan*, *Col2* and catabolism factors *Mmp13*, *Adamts5* in aging NPCs treated with Control, OKS, M-Exo and OKS@M-Exo groups. (n = 4/group). (B) WB analysis of ADAMTs5 and MMP13 in aging NPCs treated with Control, OKS, M-Exo and OKS@M-Exo groups. β-actin served as the loading control. *P < 0.05, ***P < 0.001, ****P < 0.0001, NS means none sense, one-way ANOVA with Tukey's multiple comparisons.

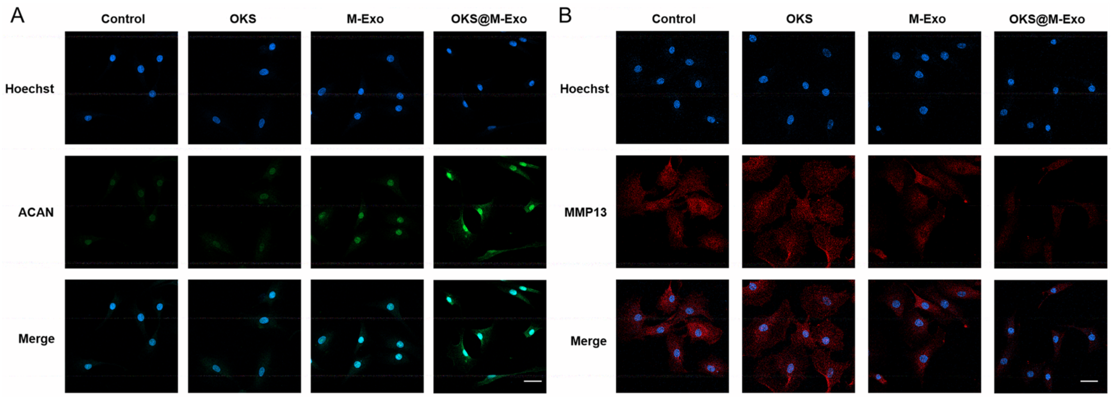

Fig. S16. Aging NPCs treated with OKS@M-Exo increased the expression of ACAN and decreased the expression of MMP13. (A,B) Representative confocal immunofluorescence micrographs showing the expression of ACAN (A) and MMP13 (B) (green) in aging NPCs treated with Control, OKS, M-Exo and OKS@M-Exo groups. Scale bar, 50 μm.

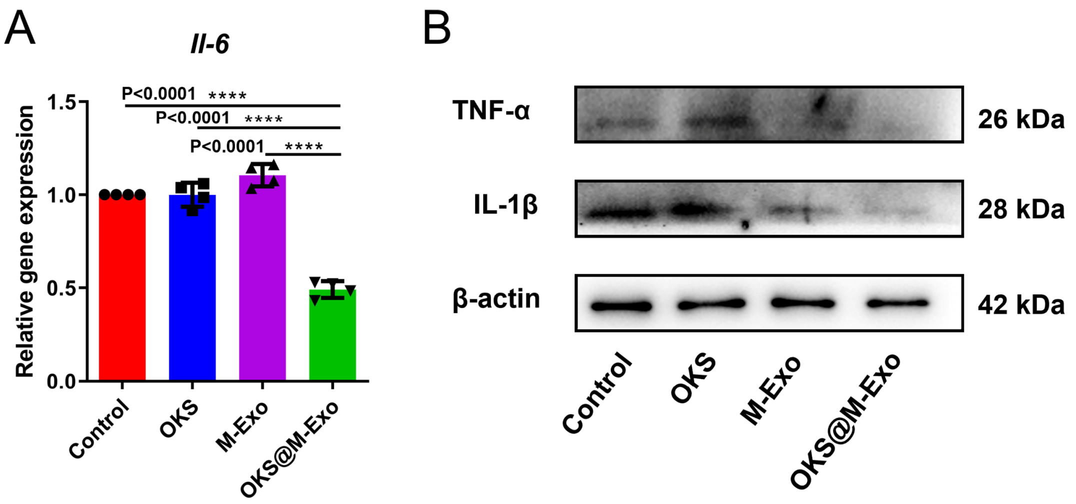

Fig. S17. Aging NPCs treated with OKS@M-Exo resulted in downregulated expression of inflammatory factor. (A) RT-qPCR analysis of senescence-associated inflammatory factors *Il-6* in aging NPCs treated with Control, OKS, M-Exo and OKS@M-Exo groups. (n = 4/group). (B) WB analysis of TNF-α and IL-1β in aging NPCs treated with Control, OKS, M-Exo and OKS@M-Exo groups. β-actin served as the loading control. ****P < 0.0001, one-way ANOVA with Tukey's multiple comparisons.

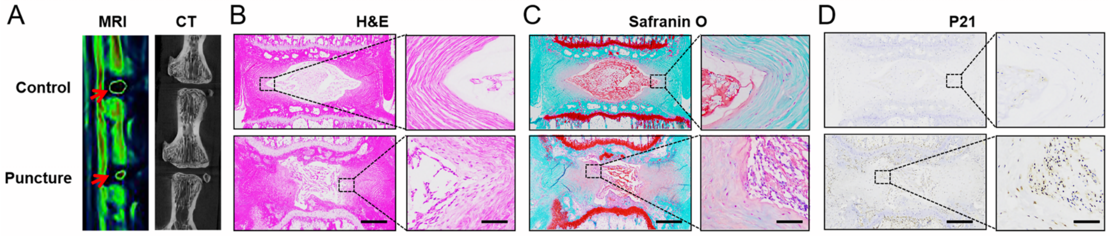

Fig. S18. The needle puncture in rat coccygeal discs could simulate the pathological process of IVDD and lead to signs of IVD aging. (A) Representative T2-weighted MRI (left) and Micro-CT (right) sagittal images of coccygeal IVD in rats at 2 weeks post puncture (The red arrow indicated the IVD). (B) Representative H&E staining images of IVD in Control and puncture groups at weeks 2 post puncture. Black boxes indicate the area shown at ×30 magnification in images right. Scale bar, 500 μm (left), scale bar, 50 μm (right). (C) Representative Safranin O-Fast Green Staining images of IVD in Control and puncture groups at weeks 2 post puncture. Black boxes indicate the area shown at ×30 magnification in images right. Scale bar, 500 μm (left), scale bar, 50 μm (right). (D) Representative IHC analysis of rat IVD sections to assess P21 in Control and puncture groups at weeks 2 post puncture. Black boxes indicate the area shown at ×30 magnification in images right. Scale bar, 500 μm (left), scale bar, 50 μm (right).

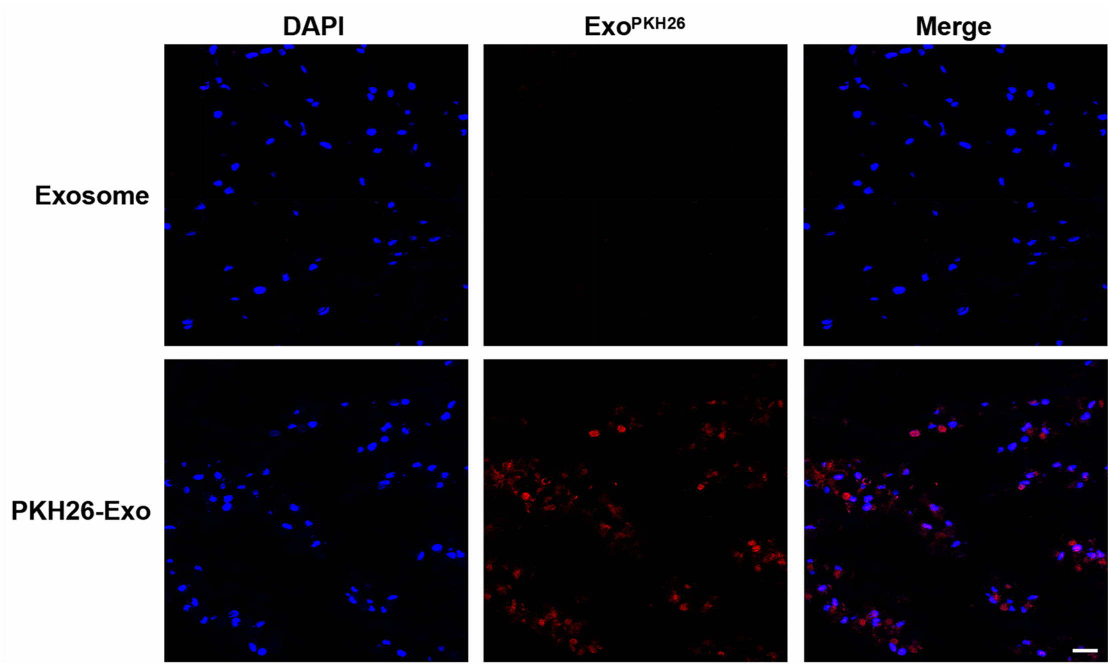

Fig. S19. OKS@M-Exo were endocytosed by nucleus pulposus cells *in vivo*. Fluorescence microscope observation of the nucleus pulposus tissue sections treated with unlabeled exosomes (Exosome) or PKH26-labeled exosomes (PKH26-Exo). Scale bar, 20 μm.

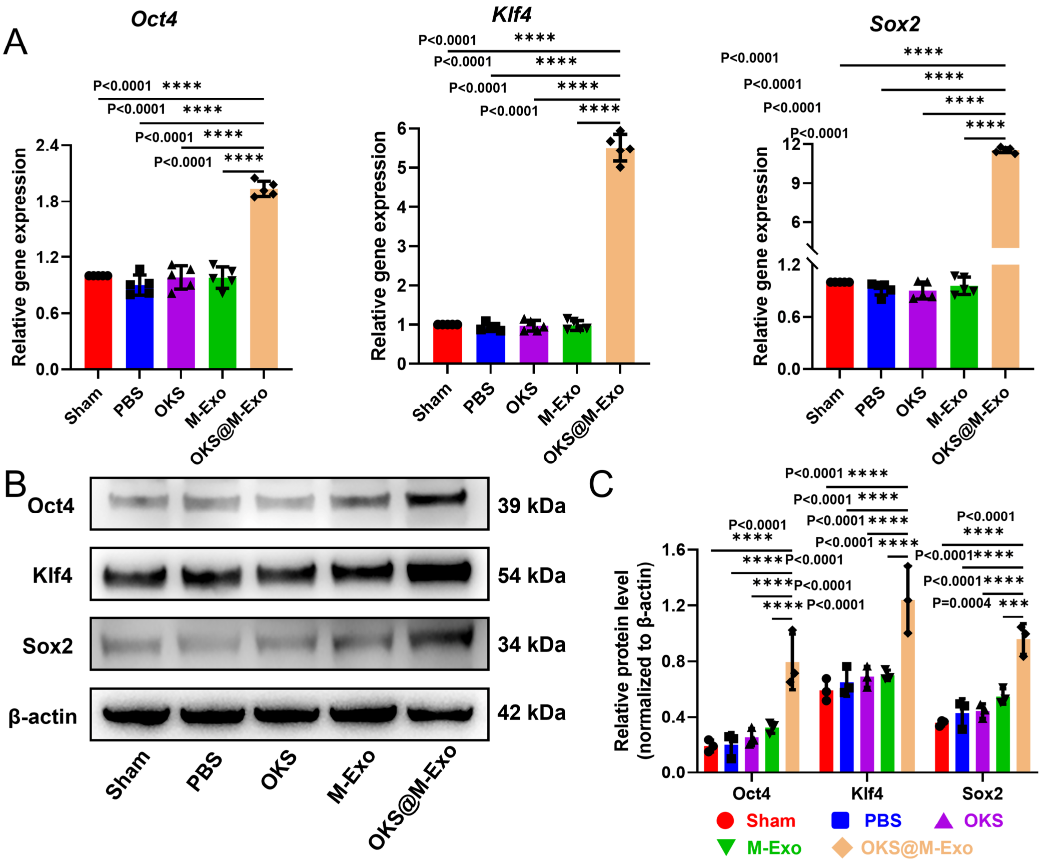

Fig. S20. Specific expression of Oct4, Klf4, and Sox2 in disc tissue after administration of OKS@M-Exo. (A) RT-qPCR analysis of *Oct4*, *Klf4*, and *Sox2* in Sham, PBS, OKS, M-Exo, and OKS@M-Exo groups. (****P < 0.0001, NS means none sense, one-way ANOVA with Tukey's multiple comparisons, n = 5/group). (B,C) WB analysis (B) and semi-quantification (C) of Oct4, Klf4, and Sox2 in Sham, PBS, OKS, M-Exo, and OKS@M-Exo groups. (n = 3/group). ***P < 0.001, ****P < 0.0001, one-way ANOVA with Tukey's multiple comparisons.

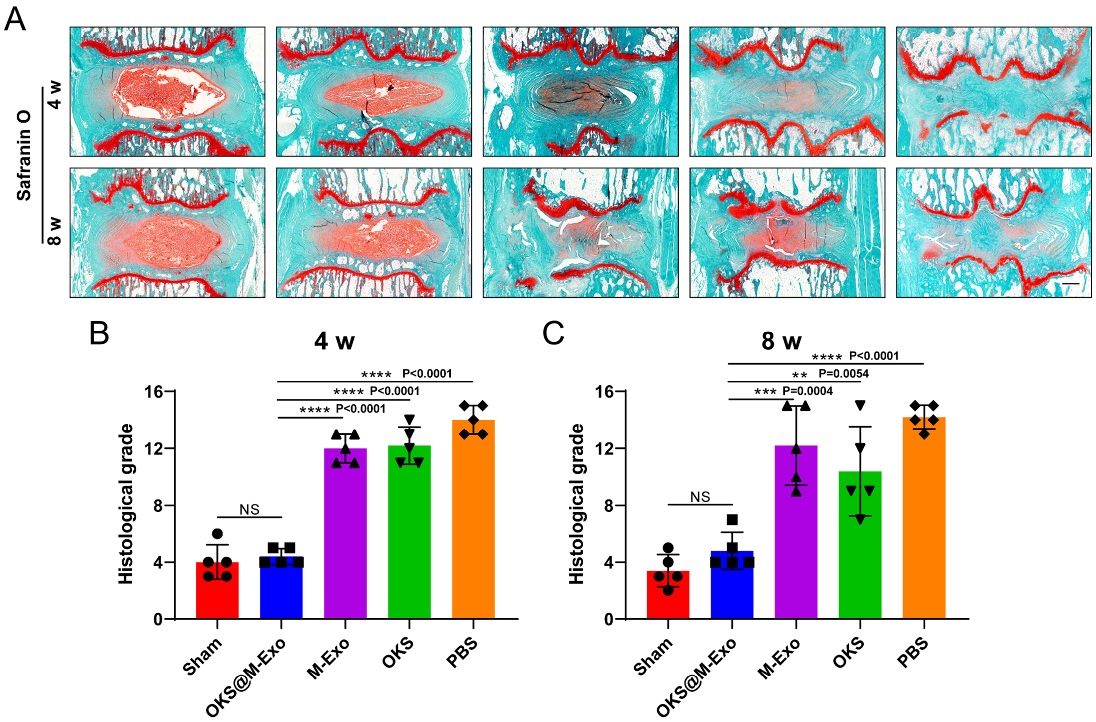

Fig. S21. Histological results of animal experiments. (A) Representative Safranin O-Fast Green Staining images of IVD in Sham, PBS, OKS, M-Exo and OKS@M-Exo groups at weeks 4 and 8 after treatment. Scale bar, 500 μm. (B-C) The histological grade of Sham, PBS, OKS, M-Exo and OKS@M-Exo groups at weeks 4 (B) and 8 (C) after treatment. (**P < 0.01, ***P < 0.001, ****P < 0.0001, NS means none sense, one-way ANOVA with Tukey's multiple comparisons, n = 5/group). Data are presented as the mean ± SD.

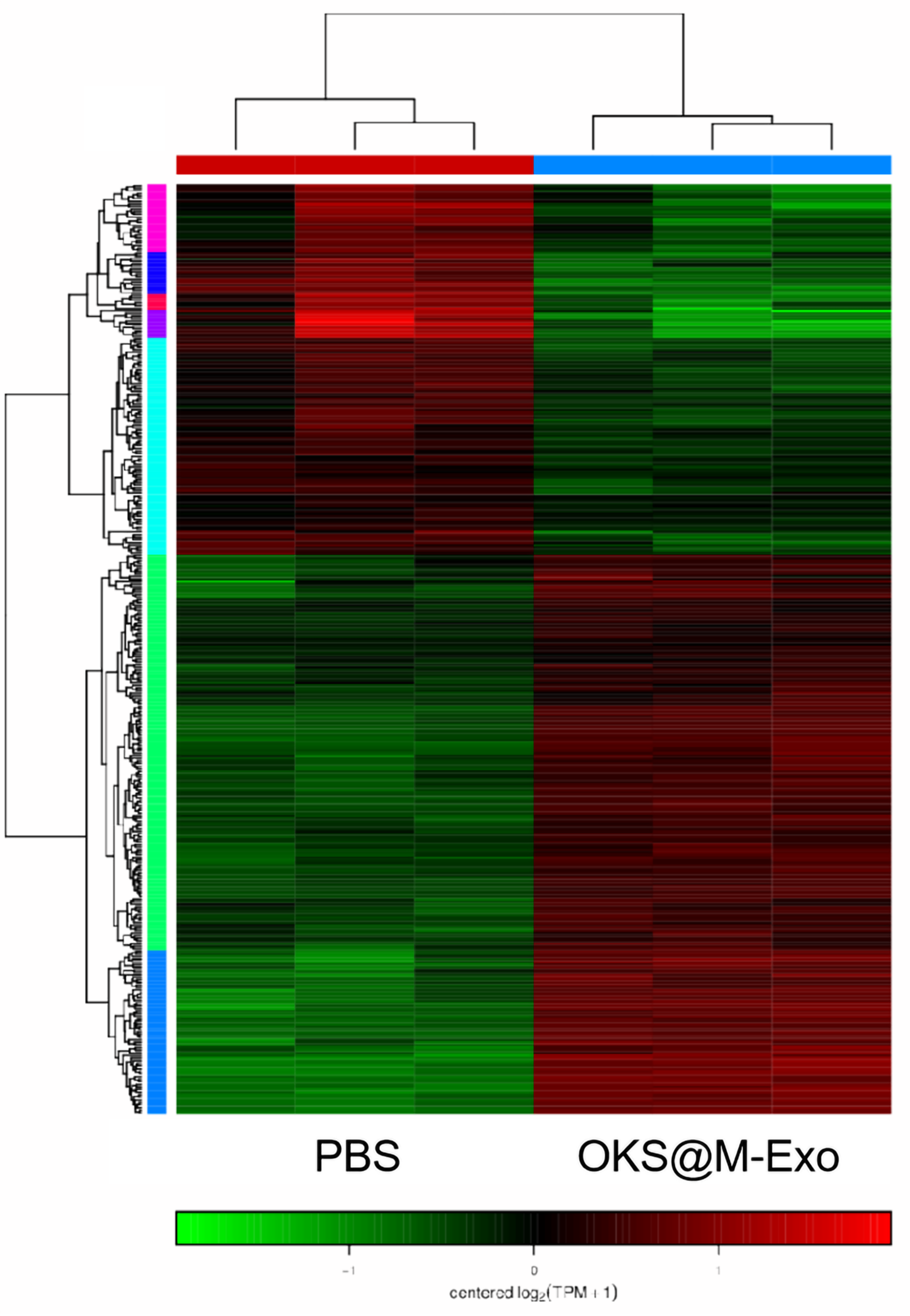

Fig. S22. The RNA-seq analysis between the OKS@M-Exo and PBS groups. Heatmap showing the relative expression levels of DEGs in PBS and OKS@M-Exo groups.

Supporting tables

Table S1. Primers used for RT-qPCR

Gene	Primer sequence
Oct4 F	TGGGGCCTAGATTCTTTTACTG
Oct4 R	AGAACTTAAGAACTTCGGACGG
Klf4 F	CATCAGTGTTAGCAAAGGAAGC
Klf4 R	TTGCTTAATCTTGGGGCACATA
Sox2 F	AAACTAATCACAACAATCGCGG
Sox2 R	GCTCCGTCTCCATCATGTTATA
P16 F	GGAGTCCTCTGCAGATAGACTA
P16 R	TGCCAATCATCATCACCTGTAT
P21 F	CAGACCAGCCTAACAGATTTCT
P21 R	AGACACACTGAATGAAGGCTAA
P53 F	CCTTACCATCATCACGCTGGAAGAC
P53 R	AGGACAGGCACAAACACGAACC
Atf3 F	CCTCTCACCTCCTGGGTCACTG
Atf3 R	TGCTTGTTCTGGATGGCGAATCTC
Gadd45b F	TGCCTCCTGGTCACGAACTGTC
Gadd45b R	CCACTGGTTATTGCCTCTGCTCTC
Col2 F	ACGCTCAAGTCGCTGAACAAC
Col2 R	AATCCAGTAGTCTCCGCTCTTCC
ACAN F	TATGATGTCTACTGCTACGTGG
ACAN R	GTAGAGGTAGACAGTTCTCACG
MMP13 F	AGCTCCAAAGGCTACAACTTAT
MMP13 R	GTCTTCATCTCCTGGACCATAG
ADAMTs5 F	CTCCTCTTGGTGGCTGACTCTTC
ADAMTs5 R	GGTTCTCGATGCTTGCATGACTG
IL-6 F	CTTCCAGCCAGTTGCCTTCTTG
IL-6 R	TGGTCTGTTGTGGGTGGTATCC
IL-1β F	ATGCCTCGTGCTGTCTGACC
IL-1β R	TTTGTCGTTGCTTGTCTCTCCTTG
β-actin F	AGATCAAGATCATTGCTCCTCCT
β-actin R	ACGCAGCTCAGTAACAGTC

Table S2. Histological grade scale of intervertebral disc

I. Nucleus pulposus morphology	II. Nucleus pulposus cellularity
Grade:	Grade:
0: round shape, nucleus pulposus > 75% of disc area	0: stellar-shaped cells
1: round shape, nucleus pulposus = 50% - 75% of disc area	1: most stellar-shaped cells with some round cells
2: nucleus pulposus = 25% -50% of disc area	2: most round cells with some stellar-shaped cells
3: nucleus pulposus < 25% of disc area	3: round-shaped cells
**III. Annulus fibrosus morphology**	**IV. Annulus fibrosus cellularity**
Grade:	Grade:
0: well-organized lamellae with no ruptures	0: fibroblasts > 90% of the cells
1: ruptured fibers < 25% of the annulus fibrosus	1: fibroblasts = 75% - 90% of the cells
2: ruptured fibers = 25% - 50% of the annulus fibrosus	2: fibroblasts = 25% - 75% of the cells
3: ruptured fibers > 50% of the annulus fibrosus	3: fibroblasts < 25% of the cells
**V. Border between the nucleus pulposus and annulus fibrosus**	
Grade:	
0: normal, without any interruption	
1: minimal interruption	
2: moderate interruption	
3: severe interruption	
